# Chelating Silicone Dendrons: Trying to Impact Organisms by Disrupting Ions at Interfaces

**DOI:** 10.3390/molecules27061869

**Published:** 2022-03-14

**Authors:** Miguel Melendez-Zamudio, Kevina Chavda, Michael A. Brook

**Affiliations:** Department of Chemistry and Chemical Biology, McMaster University, 1280 Main St. W., Hamilton, ON L8S 4M1, Canada; melendem@mcmaster.ca (M.M.-Z.); chavdk1@mcmaster.ca (K.C.)

**Keywords:** silicone dendrons, chelation, cysteamine, mercaptopropionic acid, 2-, 3-, 4-fold symmetry, ionic crosslinking, disrupting pathogens

## Abstract

The viability of pathogens at interfaces can be disrupted by the presence of (cationic) charge and chelating groups. We report on the synthesis of silicone dendrimers and linear polymers based on a motif of hexadentate ligands with the ability to capture and deliver metal ions. Mono-, di- or trialkoxysilanes are converted in G1 to analogous vinylsilicones and then, iteratively using the Piers-Rubinsztajn reaction and hydrosilylation, each vinyl group is transformed into a trivinyl cluster at G2. The thiol-ene reaction with cysteamine or 3-mercaptopropionic acid and the trivinyl cluster leads to hexadentate ligands 3 × N–S or 3 × HOOC–S. The compounds were shown to effectively capture a variety of metals ions. Copper ion chelation was pursued in more detail, because of its toxicity. On average, metal ions form chelates with 2.4 of the three ligands in a cluster. Upon chelation, viscous oils are converted to (very) soft elastomers. Most of the ions could be stripped from the elastomers using aqueous EDTA solutions, demonstrating the ability of the silicones to both sequester and deliver ions. However, complete ion removal is not observed; at equilibrium, the silicones remain ionically crosslinked.

## 1. Introduction

The evolution of COVID-19 has amplified the need to better control the viability of pathogens and particularly their longevity on surfaces. The use of high-density cationic charge is a common strategy for killing bacteria on surfaces [1,2,3]. This typically arises from amine-modified surfaces that are cationic at normal pH and, of course, quaternary ammonium surfactants are well known for their utility as generic disinfectants, including at biological interfaces [4].

**Figure 1:**
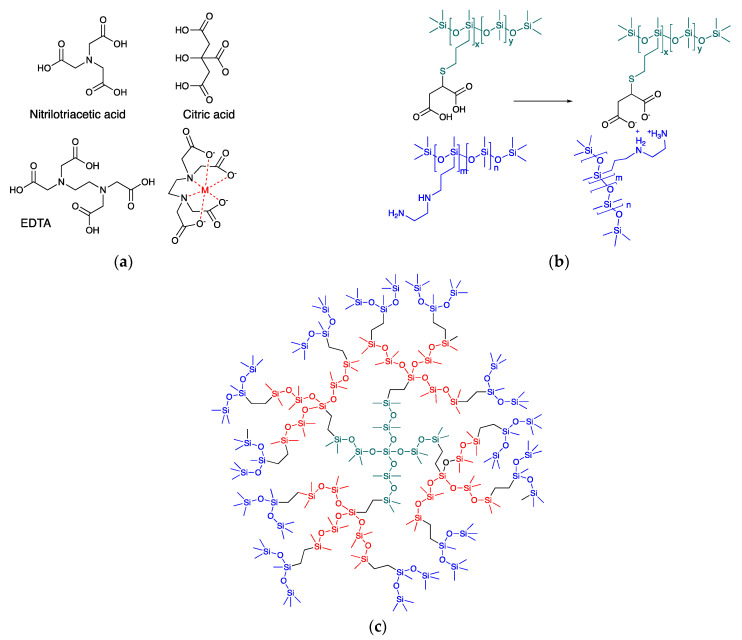
(**a**) Traditional chelating multidentate ligands. (**b**) Silicone elastomers crosslinked through ionic interactions [24,25]. (**c**) Example of a dendrimer prepared using the PR reaction [30,31].

Several papers have noted that the viability of viruses may be compromised by the presence of chelating agents [5,6,7]. Chelating groups are polydentate ligands that act as pincers that surround (specific) metal ions (Figure 1a). The process is widely used in applications ranging from the mundane, such as their use to reduce water hardness; inclusion of EDTA in foods to aid in sequestration and then excretion of heavy metal ions [8]; to the more sophisticated use of ‘his-tags’ (oligohistidine groups that present at the end of a protein) to facilitate the separation of desired proteins by chelation with nickel-containing resins [9]. In the case of viruses, chelation has been proposed as a version of chemotherapy [10,11,12]; chelating groups compete for metal ion resources required by the organism that, ideally, lead to viral deactivation [5].

A variety of metal-chelating polymers are known in the art. Typically, chelating compounds are based on carboxylates that capture hard metal ions [13,14], or amines to capture late transition metal ions [15], or heavy metals [16]; less frequently, sulfur atoms are included to capture soft metals [17]. Winnik summarized many of these in an interesting paper that described the synthesis of polycarboxylic acids from cyclopropane dicarboxylic acid [18]. The current research examines the ability to tether chelating groups to silicone elastomers and examine their ability to compete for multivalent metal ions.

Silicone elastomers are widely used in biomedical devices, both topically and internally. The materials deliver exceptional performance because of the ease of preparation in complex forms, sterilizability, and biocompatibility—they do not elicit a strong foreign body response [19]. Somewhat counter to intuition, the best biological outcomes for biomedical devices do not necessarily involve the ability of cells to proliferate on or near an implant device. For example, the growth of cells on (silicone) intraocular lenses is associated with decreased visual acuity.

More pertinent to this paper is the observed correlation between bacterial contamination and capsular contracture, which is a common problem for patients with breast implants [20]. In this case, the surface is thought to facilitate undesired bacterial growth. We were interested in developing materials with beneficial properties noted for silicones but without the attendant ability to support cell growth and, preferably, to render ineffective pathogens, including viruses and bacteria. That is, rather than being passive to local biology, the silicone itself could be functionalized to permit the growth of desired organisms in a perfect world and the suppression of pathogens. The strategy adopted involves the use of chelating silicone polymers to create active films whose surface properties can be tuned through innate charge and/or specific chelating abilities.

One of the most common preconceptions about silicones is associated with their hydrophobicity. Although the surface energy of polymers based on fluorinated hydrocarbons is lower than that of dimethylsilicones [21], the mobility of silicones—with a T_g_ < −120 °C and a T_m_ of <−50 °C—allows them to migrate or conform to local environments at room temperature so that they convey excellent water repellency, making them effectively more hydrophobic than fluorocarbons. The incorporation of organic groups can be used to change the hydrophobic properties of methylsilicones; balancing this hydrophobicity with hydrophilic groups to create active surface species is a long-standing area of research. Note that if the hydrophilic content is sufficiently high, for example, 50% amines Me_3_Si((OSiMe_2_)_n_(OSiMe(CH_2_CH_2_CH_2_NH_2_)_n_)_m_OSiMe_3_, the polymers may even be water-soluble. The most common hydrophile used in commerce with silicones is poly- or oligo(ethylene oxide)(PEG). Silicone PEGs are used, for example, as polyurethane foam stabilizers and as agricultural adjuvants [22]. Other organic functional groups that convey surface activity to methylsilicones include amines, to give fluids that are used to modify/size fabrics, and carboxylic acids. The self-association of carboxylic acid groups in a silicone environment through hydrogen bonding dramatically increases viscosity and makes the polymers more difficult to process. However, the tunable acidity allows carboxy-modified silicones to be used as constituents of ionic liquids [23], or ionically crosslinked elastomers (Figure 1b) [24,25,26].

The benefits of dendrimeric structures that can also chelate metals are well known. Dvornic et al. described the creation of silicone-modified PAMAM dendrimers that were shown to be very efficient at metal sequestration [27]. The challenges in preparing dendrimers are also well known, particularly at higher generations. Silicones are very sensitive to acids/bases, which makes routes to precise silicones, such as dendrons, normally difficult. However, the Piers-Rubinsztajn (PR) reaction [28] is particularly conducive to the preparation of highly branched silicones with spatial control [29], including dendrimers [30,31] (Figure 1c).

There are very few reports of silicones that can chelate metals; such materials should have the possibility of affecting proximal biology. Gonzaga et al. showed that citrate-modified silicones exhibited the unusual ability to reduce gold ions and then assemble the resulting Au(0) into nanosheets of gold [32], and Stan and Brook described chelating silicones based on nitrilotriacetic acids [33]. In the latter case, it was shown that surface activity was affected by the nature of the chelated metal ion. Classic chelating moieties include EDTA, nitrilotriacetic acid, and citric acid (Figure 1a), for which the binding constants to a wide variety of metal ions are known [8]. The benefit of a dendrimeric structure is that, when suitably prepared, it should be possible to present multidentate ligands at the periphery of the polymer. We reasoned that chelating silicones would result from the combination of simple routes based on PR chemistry + hydrosilylation to create dendrimeric multifunctional vinylsilicone structures and then thiol–ene reactions to install ~SCH_2_CH_2_COOH and ~SCH_2_CH_2_NH_2_ ligands. These products were tested for their ability to capture multivalent ions; Cu^2+^ was selected as a particularly relevant example because of its toxicity [34]. Note, in this paper, a ligand refers to a single bidentate cysteamine or mercaptopropionate moiety, while a chelator refers to a cluster of THREE such ligands.

## 2. Results

### 2.1. Polymer Synthesis

**Figure 2:**
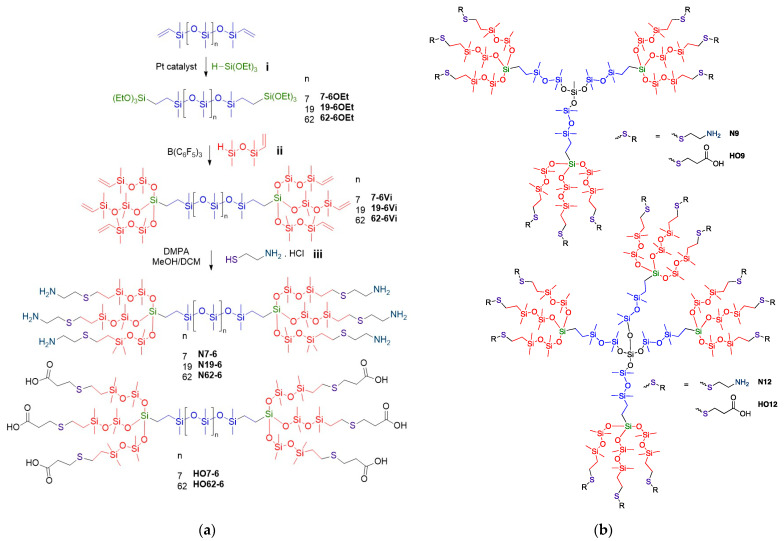
(**a**) The conversion of vinylsilicones into trivinylsilicones, and then sixfold cysteamine or mercaptopropionic acid-derived ligands. (**b**) Nine- and 12-fold analogues.

**Figure 3:**
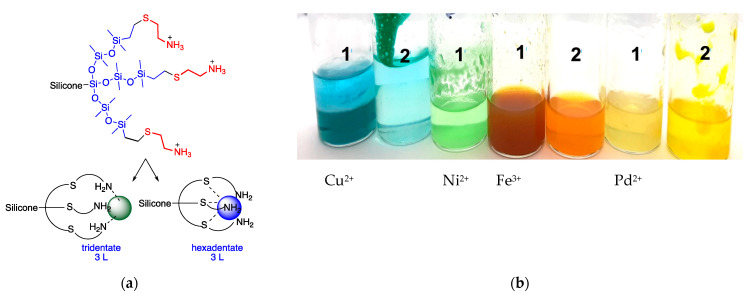
(**a**) Possible tri/hexadentate binding motifs to a tricysteamine chelator. (**b**) Chelation of amine and COOH ligands (1 = **N7-6** or 2 = **HO7-6** in DCM) to various metals salts in water (upper layer).

It is relatively straightforward to convert a single vinyl group on a silicone polymer—a class of compounds readily available in monofunctional, telechelic (α,ω-substituted), random pendent and precise pendent formats—into a trivinylsilicone [29]. To do so, two iterative steps are used to get to the next generation. The vinyl compound first undergoes a platinum-catalyzed hydrosilylation with HSi(OEt)_3_, and then, in the same pot, a Piers-Rubinsztajn reaction with a hydro, vinylsiloxane is performed (i, ii Figure 2a). The clean, high-yielding reactions, and purification are straightforward, as residual reagents have a high vapor pressure. While a trifunctional silicone was generated from RSi(OEt)_3_, leading to a tri- or hexadentate chelator (Figure 3a), it is also possible to create di/tetradentate ligands by using HMeSi(OEt)_2_.

Two classes of vinylsilicones were created: small dendrons with tri- and tetra-fold symmetry depending on starting material (i.e., 9- **9Vi** or 12-vinyl ligands **12Vi**, Figure 2b); and hexafunctional linear silicones terminated by a trivinylcluster. The latter compounds possess the same end groups but differ by the spacer length (**n-6Vi**, n = 7, 19, 62 Figure 2a).

The thiol-ene reaction was used to introduce bidentate ligands, S/N using cysteamine [35] or S/COOH using 3-mercaptopropionic acid, respectively, to the silicone dendrimers. Although attempts were made to facilitate this reaction with the thermal generation of radicals, for example using AIBN (azobisisobutryonitrile), it proved difficult to avoid undesired, competitive gelation (data not shown). The photoinitiated reactions using DMPA as initiator (2-dimethoxy-2-phenylacetophenone), by contrast, were very clean, and the products were produced in excellent yield, from 70–95% (Table 3, for selected MALDI mass and NMR spectra, see the Supplementary Materials, Figures S1–S24). Separation of the product from excess cysteamine or 3-mercaptopropionic acid was facilitated by adding water or brine or other polar solvents in which the ligand dissolved, but the silicone did not. **Nx** samples (cysteamine derivatives) presented a slightly yellow coloration, while **HOx** (3-mercaptopropionic acid derivatives) were transparent; all products were viscous oils.

### 2.2. Chelation

Survey experiments were used to establish if the ligands retained their ability to bind to metal ions once tethered to silicone dendrons. The exposure of selected silicone compounds **N7-6** or **HO7-6** in DCM (dichloromethane) to solutions containing metal ions Cu^2+^, Ni^2+^, Fe^3+^, Pd^2+^ demonstrated efficient ion capture by both cysteamine and 3-mercaptopropionic derivatives (Figure 3). More detailed studies were then undertaken with copper(II) ions, in part because its blue/green color is readily followed during migration into the silicone and because it can be toxic to pathogens; copper is increasingly used as a constituent of antifouling coatings (ultimately, we hope to test if sufficient copper is released from silicone elastomer surfaces to affect adjacent biology) [36]. Copper (II) acetate was selected because, unlike copper chloride, it is relatively soluble in organic solvents like alcohols in which silicones are partly soluble.

**Figure 4:**
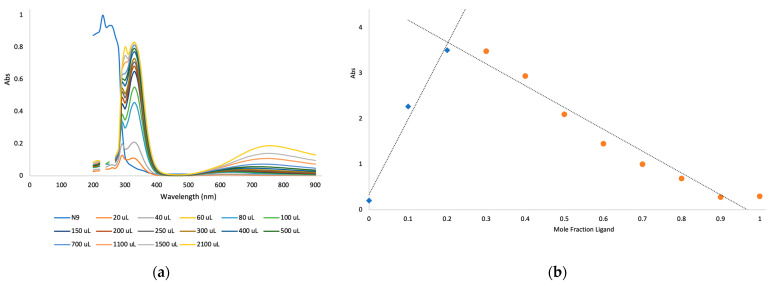
(**a**) Titration of **N9** by copper (II). (**b**) Job plot of **N9**.

Silicone ligands were dissolved in isopropanol (IPA) and then titrated with aqueous solutions of copper (II) acetate. Two features immediately became apparent: the presence of a new absorbance at 330 nm; and a drift in the maximum absorbance of Cu near 760 down to as low as 620 (Figure 4a, Figure S25), which are ascribed to metal cysteamine complexes. It was of interest to better understand the stoichiometry of the complex. One strategy to achieve this utilizes continuous variation—Job’s plot method—in which the total moles of chelating compound + copper ion is held constant while the mole ratio is systematically varied and a physical property like absorbance is measured [37]. The crossover in slope occurs at about 0.22 **N9**/Cu atom (Figure 4b). Compound **N9** bears nine cysteamine groups, which therefore translates to two cysteamine groups/Cu atoms (and one unbound). That is, at maximum binding, 2/3 of the available cysteamine groups are bound to copper. Plots for **N12** (0.2 **N12** × 12/Cu) and **N6** (0.4 **N6** × 6/Cu) both show 2.4 cysteamine groups are involved in copper ion complexation (Figure S26). Thus, these ligands cooperatively bind—chelate—to copper (II).

**Figure 5:**
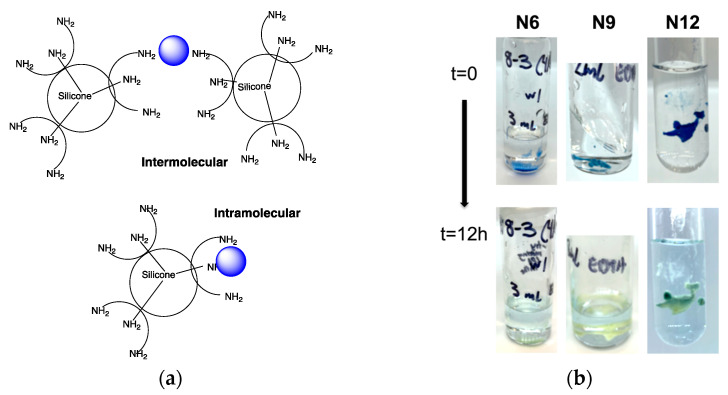
(**a**) Inter- vs. intramolecular chelation for **N9**, the former leading to crosslinks. (**b**) Transfer of ions from chelated silicones to aqueous EDTA solution over 12 h.

Initially, oils, once chelated to metals and after removal of solvents, the products were transformed into very soft gel-like materials or elastomers. This indicates that, in addition to multidentate intramolecular chelation (Figure 3a), some ions are bridging different molecules. This effect was dependent on the ion concentration and the structure of the chelator. For example, **N6** formed soft gel-like structures at Cu^2+^/ligand ratios of 1.17 and 0.59. **N12** formed elastomers at 1.93 and 0.968, while **N9** was an elastomer with a 1.46 ratio, but a soft material at a 0.73 ratio (Figure 5a). These distinctions likely arise from the statistical facility for intermolecular binding, which will be higher in the order of 4- > 3- > 2-fold symmetry **N12** > **N9** > **N6**.

### 2.3. Chelation for Elastomer Surface Only Modification

We were curious if the dendrimer could be used to create an active layer on inexpensive silicone elastomers. An elastomer created using Sylgard 184 was, after curing, coated with a thin film of **HO62-2** dissolved in IPA. After evaporation, the elastomer was soaked in a 0.33 M solution of copper (II) acetate for 5 min. After drying, a UV visible spectrum showed a copper absorption only for the silicone **HO62-2** + copper, while the absorbance was absent in the controls: silicone, silicone + **HO62-2**, or silicone + copper (Figure S27).

### 2.4. Competitive Chelation with EDTA

EDTA (ethylenediaminetetraacetic acid) is widely perceived as the gold standard of chelating molecules. The ability of the different copper chelates to resist ion transfer to EDTA was tested by exposing a complexed rubber to aqueous EDTA. After about 12 h at room temperature, most of the blue color had transferred to the EDTA in water (Figure 5b). Note, however, that the silicone ligand did not revert to its original oil state. Instead, the materials were softer than when inserted into the solution but maintained their shape as (very) soft elastomers, consistent with crosslinking through ionic bridging (Figure 5a).

## 3. Discussion

Efficient chelation requires molecules in which the proximal location of multiple ligands is controlled. In the case of the prototypical chelating molecules, citric acid, nitrilotriacetic acid and EDTA, the three or four COOH ligands are bound on a relatively rigid framework that facilitates the orientation of ligands with respect to each other so that cooperative binding can take place. Frequently, more rigid materials are utilized for metal chelation, as with pincer ligands [38].

Most silicones are based on very random structures and, particularly with linear silicones, have very high dispersities *Đ*_M_. It is very difficult to spatially control functional side chains on silicone polymers. This, and their high flexibility compared to organic molecules, would disqualify them as viable chelating molecules. Countervailing this general trend is the ability to assemble very precise, highly branched structures using the Piers-Rubinsztajn (PR) reaction. Previously, this has been shown to lead to dendrimeric materials of molar mass up to 13,000 g mol^−1^, beyond which, at higher generations, errors begin to occur during synthesis [30]. In this paper, we demonstrated that the combination of PR and hydrosilylation reactions allows one to assemble 2-, 3- or 4-fold symmetry structures on a silicone backbone; thiol-ene chemistry permits conversion of hydrophobic vinyl groups into metal-binding ligands based on S, NH_2_ and COOH (Figure 2); these ligands are expected to bind both soft and hard metals.

All of the compounds have clusters of three ligands that are well adapted to forming metal chelates. While the spacing between ligands is much larger than, for example, in EDTA, the silicone backbones are much more flexible and, we argue, are able to coalesce around a given metal ion. This was shown to be the case with a variety of different metals that were readily captured from an aqueous solution into a silicone fluid (Figure 3). More detailed studies using copper using Job plots showed that for **N6** and **N12** the most efficient binding occurred at 2.4 cysteamine ligands/Cu^2+^, but of course, each ligand is bidentate, which means, on average, an approximate pentadentate binding of one Cu^2+^ for a tri-ligand cluster (for **N9**, the value was 2 × cysteamine). We infer this reflects a distribution of binding motifs that favors intra- (e.g., hexadentate) over intermolecular binding (Figure 5a). Intermolecular ionic crosslinking is occurring, as evidenced by the formation of gel-like materials and elastomers.

The multidentate silicone ligands are, in all cases, less effective chelators than EDTA, as shown by the ready transfer of most of the silicone chelated Cu^2+^ to aqueous EDTA solutions (Figure 5b). This combination of the ability to sequester metals, and then release them was the desired outcome for these silicone dendrimers. At a minimum, it should be possible to deliver metal ions like copper from chelated silicone films and then replenish them with more copper when required. More interesting will be developing an understanding of the ability of chelating groups, per se, to disrupt the viability of pathogens on silicone surfaces.

## 4. Materials and Methods

### 4.1. Materials

Hydride-terminated PDMS (**H-13-H** HMe_2_Si(OSiMe_2_)_n_OSiMe_2_H, n = 13) DMS-H03, MW = 1100 g mol^−1^; vinyl-terminated PDMS (**Vi-9-Vi** ViMe_2_Si(OSiMe_2_)_n_OSiMe_2_Vi, n = 7), DMS-V05, MW = 800 g/mol and (**Vi-64-Vi** ViMe_2_Si(OSiMe_2_)_n_OSiMe_2_Vi, n = 62) DMS-V21 (M_w_ = 6000 g/mol), vinyl-1,1,3,3-tetramethyldisiloxane, vinyldimethylethoxysilane were acquired from Gelest, Inc., (Morrisville, PA, USA) and were used as received. Triethoxysilane, tetraethoxysilane, 2-dimethoxy-2-phenylacetophenone (DMPA), cysteamine hydrochloride, cysteamine, 3-mercaptopropionic acid, platinum(0)-1,3-divinyl-1,1,3,3-tetramethyldisiloxane complex solution (Karstedt’s catalyst) in xylene (Pt 2%), triethylamine, copper (II) acetate monohydrate, nickel (II) chloride, palladium (II) acetate, sodium hydroxide, ethylenediaminetetraacetic acid disodium salt dihydrate, deuterated chloroform (CDCl_3_), deuterated methanol (MeOH-*d*_4_), chromium (III) acetylacetonate, DHB (2,5-dihydroxybenzoic acid), neutral aluminum oxide Brockmann I, activated carbon, Celite^®^ S, Sylgard 184 elastomer kit, methanol and tetrahydrofuran were purchased from Sigma-Aldrich (Oakville, ON, Canada). Tris(pentafluorophenyl)borane (BCF) was obtained from Alfa-Aesar (Ward Hill, MA, USA). EDTA (disodium salt, dihydrate) was purchased from Anachem (Montreal, QC, Canada). Commercial toluene, dichloromethane (DCM), hexanes were dried using an activated alumina column before use.

### 4.2. Analysis Methods

^1^H- and ^29^Si-NMR spectra were recorded with a Bruker AV-600 spectrometer (Milton, ON, Canada) at room temperature using CDCl_3_ as a solvent and analyzed using Bruker Topspin software. For ^29^Si-NMR, chromium (III) acetylacetonate was used as a paramagnetic relaxation agent. Infrared spectroscopy was done using a Thermo Scientific Nicolet 6700 FT-IR (Waltham, MA, USA) spectrometer using a Smart iTX attenuated total reflectance (ATR) attachment. UV−Vis spectroscopy measurements were obtained on a BioTek Synergy LX multimode reader (Santa Clara, CA, USA), recorded from 200—900 nm. Gel Permeation Chromatography was performed on a Waters 1500-series HPLC pump (Milford, MA, USA) coupled to a Waters 2414 refractive index detector; calibration was done using polystyrene calibration kit S-M-10 (Lot 85) from Polymer Laboratories; HPLC toluene was used as eluent. UV-photochemistry was performed using a High-Intensity UV Lamp by Analytik Jena (UVP-B-100AP) (Upland, CA, USA) at 365 nm and 100 watts. Short-path vacuum distillation was performed using a kugelrohr B-585 from Büchi (New Castle, DE, USA). For MALDI mass spectra (MS) were measured using a Bruker UltrafleXtreme MALDI TOF/TOF; 2 μL of compound (1 mg/mL in IPA, (isopropanol)), 10μL DHB (2,5-dihydroxybenzoic acid, 100 mg/mL in THF), 2 μL NaOAc (100 mM in MeOH/H_2_O 50:1).

### 4.3. Polymer Synthesis

#### 4.3.1. Formation of 4-Arm **4-Vi**

**4-Vi**: tetraethoxysilane (2.00 g, 9,60 mmol) was dissolved in dry hexanes (5 mL) in a dry, N_2_ purged 100 mL round-bottomed flask. B(C_6_F_5_)_3_ (0.73 mL, 5.76 µmol) was added to the stirring solution, after which vinyl-1,1,3,3-tetramethyldisiloxane (6.15 g, 38.4 mmol) was added dropwise using a syringe. The solution was allowed to stir at RT for 3 h. Aluminum oxide was added to the solution and allowed to stir for 12 h. The resulting solution was filtered via vacuum filtration through a Celite pad, and the solvent removed under reduced pressure. The resulting product was subjected to kugelrohr distillation at 125 °C to remove impurities. **4-Vi** was obtained with a yield of 97% (6.85 g). The same procedure was employed to synthesize **3-Vi** and **2-Vi**.

**4-Vi:**^1^H-NMR (CDCl_3_, 600 MHz): δ 6.14–5.70 (m, 12 H), 0.15 (s, 24H), 0.07 (s, 24H). ^29^Si-NMR (CDCl_3_, 119 MHz): δ −3.96, −20.35, −109.51 ppm.

For the remaining G1 vinyl compounds, see Table 1.

**3-Vi**: ^1^H-NMR (CDCl_3_, 600 MHz): δ 6.16–5.71 (m, 9 H), 0.16 (s, 18H), 0.07 (s, 21H). ^29^Si-NMR (CDCl_3_, 119 MHz): δ −3.93, −20.92, −67.51 ppm.

**2-Vi**: ^1^H-NMR (CDCl_3_, 600 MHz): δ 6.15–5.71 (m, 6 H), 0.15 (s, 12H), 0.07 (s, 130H). ^29^Si- NMR (CDCl_3_, 119 MHz): δ −3.93, −20.92 ppm.

#### 4.3.2. Formation of 12-Arm Alkoxysilane **12OEt**

**12OEt: 4-Vi** (2.00 g, 2.74 mmol) and triethoxysilane (2.02 g, 12.30 mmol) were added to a pre-dried 100 mL round-bottomed flask together with dry hexanes (5 mL). The reaction flask was capped and flushed with dry nitrogen. Karstedt’s catalyst (3 μL, 6.71 μmol) was then directly added from the bottle. The mixture was stirred at room temperature for 12 h at room temperature before activated carbon was added to the flask to quench the Karstedt’s catalyst, followed by filtration through a Celite pad, the solvent removed under reduced pressure. The resulting product was subjected to kugelrohr distillation at 135 °C to remove impurities. **12OEt** was obtained with a yield of 92% (3.5 g) (for other examples, see Table 2). The same procedure was used for the 6 & 9 alkoxysilanes.

**12OEt:** ^1^H-NMR (CDCl_3_, 600 MHz): δ 3.82 (q, *J* = 7.00 Hz, 24H), 1.22 (t, *J* = 7.00, 36H), 0.54 (s, 16H), 0.07 (s, 24H), 0.06 (s, 24H). ^29^Si-NMR (CDCl_3_, 119 MHz): δ 8.25, −21.17, −44.54, −109.05 ppm.

For the remaining G1 alkoxysilanes, see Table 2.

**9OEt**: ^1^H-NMR (CDCl_3_, 600 MHz): δ 3.82 (q, *J* = 7.00 Hz, 18H), 1.22 (t, *J* = 7.00, 27H), 0.55 (s, 12H), 0.068 (s, 18H), 0.05 (s, 21H) ppm. ^29^Si-NMR (CDCl_3_, 119 MHz): δ 8.32, −21.60, −44.55, −67.40 ppm.

**7-6OEt**: ^1^H-NMR (CDCl_3_, 600 MHz): δ 3.81 (q, *J* = 6.99 Hz, 12H), 1.22 (t, *J* = 7, 18H), 0.56 (s, 8H), 0.07 (s,53 H) ppm. ^29^Si-NMR (CDCl_3_, 119 MHz): δ 8.23, −21.90, −44.65 ppm.

**19-6OEt**: ^1^H-NMR (CDCl_3_, 600 MHz): δ 3.81 (q, *J* = 6.99 Hz, 12H), 1.22 (t, *J* = 7, 18H), 0.55 (s, 8H), 0.07 (s, 142H) ppm. ^29^Si-NMR (CDCl_3_, 119 MHz): δ 8.23, −4.03, −81.86, −67.78 ppm.

**62-6OEt**: ^1^H-NMR (CDCl_3_, 600 MHz): δ 3.82 (q, *J* = 6.99 Hz, 12H), 1.22 (t, *J* = 6.99, 18H), 0.56 (s, 8H), 0.07 (s,407 H) ppm. ^29^Si-NMR (CDCl_3_, 119 MHz): δ 8.24, −21.90, −44.61 ppm.

#### 4.3.3. Formation of 12-Arm Vinylsilicone **12-Vi**

**12-Vi**: **12OEt** (3.5 g, 2.52 mmol) was dissolved in dry hexanes (5 mL) in a dry, N_2_ purged 100 mL round-bottomed flask. B(C_6_F_5_)_3_ (1.55 mL, 13.18 µmol) was added to the stirring solution, after which vinyl-1,1,3,3-tetramethyldisiloxane (5.1 g, 31.5 mmol) was added dropwise using a syringe. The solution was allowed to stir at RT for 3 h. Aluminum oxide was added to the solution and allowed to stir for 12 h. The resulting solution was filtered via vacuum filtration through a Celite pad, and the solvent was removed under reduced pressure. The resulting product was subjected to kugelrohr distillation at 125 °C to remove impurities. **12-Vi** was obtained with a yield of 89% (6.62 g). The same procedure was employed to synthesize **7-6-Vi** and **9-Vi** (Table 1).

**12-Vi**: ^1^H-NMR (CDCl_3_, 600 MHz): δ 6.15—5.70 (m, 36H), 0.53—0.39 (m, 16H), 0.15 (s, 72H), 0.07 (s, 120H) ppm. ^29^Si-NMR (CDCl_3_, 119 MHz): δ 8.60, −4.10, −21.33, −67.79, −109.49 ppm. GPC: Mn = 2752, *Đ*_M_ = 1.25.

For the remaining G2 vinyl compounds, see Table 1.

**9-Vi**: ^1^H-NMR (CDCl_3_, 600 MHz): δ 6.15—5.70 (m, 27H), 0.53—0.39 (m, 12H), 0.15 (s, 54H), 0.07 (s, 93H) ppm. ^29^Si-NMR (CDCl_3_, 119 MHz): δ 8.41, −4.04, −21.19, −67.85 ppm. GPC: Mn = 2284, *Đ*_M_ = 1.18.

**7-6Vi**: ^1^H-NMR (CDCl_3_, 600 MHz): δ 6.15—5.71 (m, 18H), 0.54—0.39 (m, 8H), 0.15 (s, 36H), 0.07 (s, 89H) ppm. ^29^Si-NMR (CDCl_3_, 119 MHz): δ 8.30, −4.13, −21.29, −67.81 ppm. GPC: Mn = 2121, *Đ*_M_ = 1.14.

**19-6Vi**: ^1^H-NMR (CDCl_3_, 600 MHz): δ 6.14—5.70 (m, 18H), 0.54—0.39 (m, 8H), 0.15 (s, 36H), 0.07 (s, 178H) ppm. ^29^Si-NMR (CDCl_3_, 119 MHz): δ 8.23, −4.03, −21.86, −67.78 ppm. GPC: Mn = 2976, *Đ*_M_ =1.43.

**62-6Vi**: ^1^H-NMR (CDCl_3_, 600 MHz): δ 6.14—5.70 (m, 18H), 0.54—0.39 (m, 8H), 0.15 (s, 36H), 0.07 (s, 443H) ppm. ^29^Si-NMR (CDCl_3_, 119 MHz): δ 8.23, −4.03, −21.86, −67.78 ppm. GPC: Mn = 6188, *Đ*_M_ =1.62.

#### 4.3.4. Conversion of Vinylsilicones into Amine or Carboxylic Acid Derivatives Using the Thiol-Ene Reaction, Shown for **N12** and **HO12**

**N12**: To an oven-dried round-bottomed flask equipped with a stirrer was added cysteamine hydrochloride (0.52 g, 4.57 mmol) in a 1:3 MeOH:DCM mixture (8 mL) (for other polymers and thiols, see (Table 3). After complete dissolution of the salt (if the solution becomes foggy more of the solvent was added until clarity was reached) **12-Vi** (1.0 g, 0.34 mmol) and then the photoinitiator DMPA were added (4.3 mg, 0.016 mmol). The flasks were covered with aluminum-foil, only exposing one side to the 365 nm UV lamp (100 watts, 1.27 W/cm^2^) for a period of 90 min. The highly viscous product **N12** was dissolved in distilled water, and triethylamine (0.63 mL, 4.57 mmol) was added. Then, the chelator was extracted from the aqueous solution using DCM, washed with brine and dried over Na_2_SO_4_. After filtration, the solution was concentrated by rotatory evaporation and dried for 24 h a N_2_ gas flow to give 0.916 g of the product in (70%) yield.

**N12**: ^1^H-NMR (CDCl_3_, 600 MHz): δ 2.86 (mt, 24H, *J* = 7.1), 2.62–2.60 (t, 24H, *J* = 7.1), 2.57–2.54 (t, 24H, *J* = 17.2), 1.51 (s, 24H), 0.91–0.88 (t, 24H, *J* = 17.4), 0.51–0.37 (m, 16H), 0.11 (s, 72H), 0.07 (s, 120H).

**N9**: ^1^H-NMR (CDCl_3_, 600 MHz): δ 2.86 (m, 18H), 2.62–2.60 (t, 18H, *J* = 7.1), 2.57–2.54 (t, 18H, *J* = 17.4), 1.48 (s, 18H), 0.91–0.88 (t, 18H, *J* = 17.5), 0.52–0.38 (m, 12H), 0.11 (s, 54H), 0.07 (s, 93H).

**N7-6**: ^1^H-NMR (CDCl_3_, 600 MHz): δ 2.86 (t, 12H, *J* = 7.1), 2.63–2.61 (t, 12H, *J* = 7.1), 2.57–2.54 (t, 12H, *J* = 17.5), 1.50 (s, 12H), 0.92–0.89 (t, 12H, *J* = 17.5), 0.51–0.39 (m, 8H), 0.11 (s, 36H), 0.70 (s, 93H).

**N19-6**: ^1^H-NMR (CDCl_3_, 600 MHz): δ 2.87–2.85 (t, 12H, *J* = 6.4), 2.63–2.61 (t, 12H, *J* = 6.3), 2.57–2.54 (t, 12H, *J* = 17.5), 1.54 (s, 12H), 0.92–0.89 (t, 12H, *J* = 17.5), 0.51–0.39 (m, 8H), 0.11 (s, 36H), 0.06 (s, 221H).

**N62-6**: 2.86 (t, 12H, *J* = 7.1), 2.63–2.61 (t, 12H, *J* = 7.1), 2.57–2.54 (t, 12H, *J* = 17.5), 1.51 (s, 12H), 0.92–0.89 (t, 12H, *J* = 17.5, 0.52–0.39 (m, 8H), 0.11 (s, 36H), 0.70 (s, 443H).

**HO12**: To an oven-dried round-bottomed flask equipped with a stirrer was added 3-mercaptopropionic acid (0.45 g, 4.23 mmol) in DCM (5 mL) (for other polymers and thiols, see Table 3). After dissolution, **12-Vi** (1 g, 0.33 mmol) and then the photoinitiator DMPA were added (4.3 mg, 0.01 mmol). The flasks were covered with aluminum foil, only exposing one side to the 365 nm UV lamp (100 watts, 1.27 W/cm^2^) for a period of 45 min. The highly viscous product **HO12** was dissolved in toluene and washed three times with water and brine. After extraction, the solution was concentrated by rotatory evaporation and dried for 24 h in a N_2_ gas flow to give 1.36 g of the product in (95%) yield. F

**HO12**: ^1^H-NMR (MeOH-*d*_4_, 600 MHz): δ 2.80–2.77 (m, 24H), 2.66–2. 58 (m, 48H), 0.91–0.88 (m, 24H), 0.51–0.39 (m, 16H), 0.11 (s, 72H), 0.07 (s, 120H). MALDI MS. Calc. for C_132_H_316_O_56_S_12_Si_37_ *m*/*z* 4217 shows a cluster of peaks centered at 4270 and doubly charged species near 2068 (Figure S1, Supplementary Materials).

For the remaining G1 amines and acids, see (Table 3).

**HO9**: ^1^H-NMR (MeOH-*d*_4_, 600 MHz): δ 2.80–2.78 (m, 18H), 2.66–2.59 (m, 36H), 0.91–0.88 (m, 18H), 0.51–0.39 (m, 12H), 0.11 (s, 53H), 0.07 (s, 93H). MALDI MS. Calc. for C_100_H_240_O_42_S_9_Si_28_ *m*/*z* 3184.77 shows a cluster of peaks centered at 3243 (Figure S1, Supplementary Materials).

**HO7-6**: ^1^H-NMR (MeOH-*d*_4_, 600 MHz): δ 2.80–2.78 (t, 12H, *J* = 7.08), 2.66–2.64 (t, 12H, *J* = 7.08), 2.62–2.59 (t, 12H, *J* = 17.46), 0.91–0.88 (t, 12H, *J* = 17.47), 0.51–0.39 (m, 8H), 0.11 (s, 36H), 0.07 (s, 93H) IR cm^−1^ 1719. MALDI MS. Calc. for C_74_H_182_O_31_S_6_Si_22_ *m*/*z* 2374.59 shows a cluster of peaks centered at 2604 (Figure S1, Supplementary Materials).

**HO62-6**: ^1^H-NMR (CDCl_3_, 600 MHz): δ 2.80–2.78 (t, 12H, *J* = 7.06), 2.66–2.64 (t, 12H, *J* = 7.06), 2.62–2.59 (t, 12H, *J* = 7.83), 0.91–0.88 (t, 12H, *J* = 17.48), 0.51–0.39 (m, 8H), 0.11 (s, 36H), 0.06 (s, 443H) ppm. IR cm^−1^ 1719.

### 4.4. Chelation Survey Experiments

Different quantities of metal salts were weighed into 10 mL vials and dissolved in 2 mL of solvent (Table 4). A silicone ligand (1 = **N7-6** or 2 = **HO7-6**, 10 mg) was pre-dissolved in DCM (50 µL). The DCM chelator solution was separately added to vials containing the salt and shaken. Photographs were taken after the two phases had completely separated (about 5 min, Figure 3b).

### 4.5. Titrations with Copper(II) Shown for ***N9***

**N9** (0.025 g, 8.5 μmol) was dissolved in IPA (5 mL) with sonication. The solution (400 μL, 0.68 μmol) was placed in each well of a 24-well plate. The solution was titrated with copper (II) acetate solution (0.025 M) in aliquots (20 μL, 0.5 μmol) until a total of 2100 μL had been added. A UV-Vis spectrum was taken after each addition (200–900 nm, 10 nm steps, Figure 4). The data for N12 and N6 is found in Figure S25, Supplementary Materials. Note the growth of the peak at 330 nm and a shift from 780 nm shifts down to around 620–680 nm.

### 4.6. Job Plots, Shown for ***N9***

**N9** (0.10 g, 34.1 μmol) was dissolved in IPA (3 mL) and sonicated. This solution (500 μL, 5.7 μmol) was used as the “100% **N9**”. The remaining measurements by fixing the total moles of reagents at 5.7 μmol, but systematically use lower quantities of **N9** solution and higher quantities of aqueous copper (II) acetate solution (0.0114 g, 0.0571mmol) in water (5 mL) to give a 11.41 M solution (Figure 4b).

**N7-6** (0.15 g, 0.0638 mmol); fixed moles 63.75 μmol; Cu(OAc)_2_ (0.0127 g, 0.0636 mmol) in water (5 mL) to give a 12.72 mM solution. **N12** (0.760 g, 0.196 mmol); fixed moles 196.08 μmol, Cu(OAc)_2_ (0.0065 g, 0.0325 mmol) in water (5 mL) to give a 6.5 mM solution (Figure S26, Supplementary Materials).

### 4.7. Ionic Crosslinking, Shown for ***N12***

**N12** (0.1 g, 26 μmol) was dissolved in 5 mL IPA. Copper (II) acetate (1 mL, 0.05 M, 50 μmol) was added and the solvents were removed using a stream of air to give a blue elastomer. A similar method with a more dilute copper (II) acetate solution (1 mL, 0.025 M, 25 μmol) gives a softer elastomer.

The process was repeated with **N9** (0.1 g, 34 μmol) to give an elastomer from the 0.05 M copper solution and a soft, gel like material for the lower concentration 0.025 Mcopper acetate. The process was repeated with **N6** (0.1 g, 43 μmol), which led to soft gel-like materials from either copper solution.

### 4.8. Thin Film Coatings of HO62-6 Absorb Copper Acetate from Water

Small circles of Sylgard 184 elastomer, cured following manufacturer’s instructions, with a diameter of 1.1 cm (thickness of 2 mm) were punched from an elastomer film and modified with the chelator **HO62-6**. A solution of the chelator **HO62-6** (0.1 g, 0.0153 mmol) was dissolved in isopropanol (1.5 mL), and drops of the solution (200 µL) were placed on a clean elastomer surface that was placed in an oven at 50 °C for 10 min to remove the IPA. The modified surface was exposed to 1.5 mL of an aqueous solution of copper (II) acetate (0.1 g, 0.5 mmol in 1.5 mL of H_2_O_dist_.) for 5 min. After exposure, the elastomer surface was dried gently using a Kimwipe and placed in the oven at 50 °C for 10 min. The same protocol was used for Sylgard (i) as a control (ii) after exposure to copper, but not **HO62-6**, and (iii) the **HO62-6**/Sylgard that was not exposed to copper. All four samples were characterized using UV-Vis spectroscopy (Figure S27, Supplementary Materials).

### 4.9. Competitive Chelation Using EDTA

Disodium, dihydrate EDTA (1.0 g, 26.9 mmol) was dissolved in 100 mL of distilled water. Small samples (~ 300 mg) of copper crosslinked ligands were placed in the EDTA solution (~3 mL) and left overnight at room temperature. In all three cases: **N6**, **N9**, **N12**, the aqueous EDTA solution had removed essentially all the blue copper coloration leaving slightly yellow silicone cysteamine elastomers. The silicone products did not return to viscous oils, but remained as soft elastomers, consistent with ionic crosslinking (Figure 5).

## 5. Conclusions

Silicone polymers are widely used as biomaterials because of their high level of biocompatibility. Organisms require an appropriate mix of metal ions to propagate. We have demonstrated that silicones can be active rather than passive biomaterials by controlling their ability to affect local ion concentrations. Dendimeric structures with 6, 9, or 12 arms and terminated with either mercaptopropionic acid or cysteamine-derived groups were readily assembled using a combination of hydrosilylation, the Piers-Rubinsztajn reaction, and thiol-ene chemistry. The compounds readily bound a variety of metals, but that binding was competitive with other good ligands, such as EDTA. The surfaces of such materials should be viable treatments for specific organisms.

## Figures and Tables

**Table 1:** Quantities used to prepare vinylsilicones from alkoxysilanes.

**G1**	**B(C_6_F_5_)_3_ mL** **(µmol)**	**Alkoxysilane and Quantity g (mmol)**	**ViSiMe_2_OSiMe_2_H g (mmol)**	**Yield g (%)**
**2-Vi**	0.276 (2.156)	DMS-H03 2 (2.69)	0.70 (5.39)	1.95 (93)
**3-Vi**	1.67 (13.44)	(EtO)_3_SiH 2.0 (11.2)	6.29 g (39.3)	6.29 (98)
**4-Vi**	0.73 (5.76)	(EtO)_4_Si 2.0 (9.60)	6.15 g (38.4)	6.85 (97)
**G2**				
**9-Vi**	3.47 (40.32)	**9OEt** 8.2 (7.72)	11.76 (73.4)	16.01 (93)
**12-Vi**	1.55 (13.18)	**12OEt** 3.5 (2.52)	5.1 (31.5)	6.62 (89)
**7-6-Vi**	2.75 (21.51)	**7-6OEt** 6.18 (5.97)	6.22 (38.8)	9.75 (90)
**19-6-Vi**	0.31 (2.48)	**18-6OEt** 1.99 (1.03)	1.03 (6.42)	2.46 (88)
**62-6-Vi**	0.649 (5.07)	**62-6OEt** 7.21 (1.41)	1.58 (9.87)	7.96 (96)

**Table 2:** Quantities used to prepare trialkoxysilicones from vinylsilicones.

	Karstedt’s (mL) (mmol)	Vinylsilicone and Quantity g (mmol)	(EtO)_3_SiH g (mmol)	Yield g (%)
**9OEt**	4.0 (8.97)	**3-Vi** 5.22 (9.17)	5.27 (32.1)	8.2 (96)
**12OEt**	3.0 (6.71)	**4-Vi** 2.00 (2.74)	2.02 (12.3)	3.5 (92)
**7-6OEt**	4.0 (8.97)	DMS-V05 5.00 (7.08)	2.91 (17.7)	6.18 (85)
**19-6OEt**	3.0 (6.71)	**2-Vi** 1.95 (1.22)	0.50 (3.05)	1.99 (85)
**62-6OEt**	7.0 (12.8)	DMS-V21 7.00 (1.46)	0.6 (3.65)	7.21 (96)

**Table 3:** Quantities used to prepare cyteine- and 3-mercaptopropanoic acid-derived silicones from vinylsilicones.

**Chelator N**	**DMPA** **mg (mmol)**	**Cysteamine g (mmol)**	**Vinylsilicone g (mmol)**	**Yield g (%)**
**N12**	4.34 (0.016)	0.52 (4.57)	**12-Vi** 1.0 (0.34)	0.91 (70%)
**N9**	5.73 (0.022)	0.48 (4.25)	**9-Vi** 1.02 (0.44)	0.91 (70%)
**N7-6**	7.05 (0.027)	0.394 (3.46)	**7-6 Vi** 1.02 (0.55)	1.08 (86%)
**N19-6**	2.68 (0.010)	0.15 (1.35)	**19-6 Vi** 0.5 (0.20)	0.49 (75%)
**N62-6**	1.73 (6.7 × 10^−2^)	0.10 (0.88)	**62-6 Vi** 0.8 (0.13)	0.73 (73%)
**Chelator OH**	**DMPA**	**HS(CH_2_)_2_COOH**	**Vinylsilicone g (mmol)**	**Yield g (%)**
**HO12**	4.34 (0.016)	0.45 (4.23)	**12-Vi** 1.01 (0.33)	1.36 (95%)
**HO9**	5.73 (0.022)	0.45 (4.25)	**9-Vi** 1.02 (0.44)	1.19 (84%)
**HO7-6**	10 (0.041)	0.56 (5.36)	**7-6 Vi** 1.5 (0.82)	1.92 (94%)
**HO62-6**	4.34 (0.016)	0.22 (2.18)	**62-6 Vi** 2.00 (0.33)	1.95 (88%)

**Table 4:** Chelation experiments for different salts.

Chelator	Salt (mg)	Solvent (2 mL)	Chelation Time after Shaken
1	Cu(OAc)_2_ (50)	H_2_O_dist_	<1 min
2	Cu(OAc)_2_ (50)	H_2_O_dist_	<1 min
1	NiCl_2_ (50)	H_2_O_dist_./MeOH (1:1)	2 days
1	FeCl_3_ (30)	H_2_O_dist_.	2 days
2	FeCl_3_ (30)	H_2_O_dist_.	2 days
1	Pd(OAc)_2_ (15)	DCM	<1 min
2	Pd(OAc)_2_ (15)	DCM	<1 min

## Data Availability

Not applicable.

## Supplementary Materials

The following are available online at https://www.mdpi.com/article/10.3390/molecules27061869/s1. Figure S1. MALDI mass spectra (Bruker UltrafleXtreme MALDI TOF/TOF; 2 μL oligomer (1 mg/mL in IPA, (isopropanol)), 10 μL DHB (2,5-dihydroxybenzoic acid, 100 mg/mL in THF), 2 μL NaOAc (100 mM in MeOH/H_2_O 50:1) 1 μL mixture loaded to target, allowed to dry.) of (A) HO12, (B) HO12, and (C) HO7-6. Figure S2. ^1^H-NMR for 2-Vi in CDCl_3_. Figure S3. (A) ^1^H-NMR and (B) ^29^Si-NMR for 3-Vi in CDCl_3_. Figure S4. (A) ^1^H-NMR and (B) ^29^Si-NMR for 4-Vi in CDCl_3_. Figure S5. ^1^H-NMR for 7-6OEt in CDCl_3_. Figure S6. (A) ^1^H-NMR and (B) ^29^Si-NMR for 19-6OEt in CDCl_3_. Figure S7. (A) ^1^H-NMR and (B) ^29^Si-NMR for 62-6OEt in CDCl_3_. Figure S8. (A) ^1^H-NMR and (B) ^29^Si-NMR for 9OEt in CDCl_3_. Figure S9. (A) ^1^H-NMR and (B) ^29^Si-NMR for 12OEt in CDCl_3_. Figure S10. (A) ^1^H-NMR and (B) ^29^Si-NMR for 7-6Vi in CDCl_3_. Figure S11. (A) ^1^H-NMR and (B) ^29^Si-NMR for 19-6OVi in CDCl_3_. Figure S12. (A) ^1^H-NMR and (B) ^29^Si-NMR for 62-6Vi in CDCl_3_. Figure S13. (A) ^1^H-NMR and (B) ^29^Si-NMR for 9Vi in CDCl_3_. Figure S14. (A) ^1^H-NMR and (B) ^29^Si-NMR for 12Vi in CDCl_3_. Figure S15. (A) ^1^H-NMR and (B) ^29^Si-NMR for N 7-6 in CDCl_3_. Figure S16. ^1^H-NMR for N 19-6 in CDCl_3_. Figure S17. ^1^H-NMR for N 62-6 in CDCl_3_. Figure S18. (A) ^1^H-NMR and (B) ^29^Si-NMR for N9 in CDCl_3_. Figure S19. (A) ^1^H-NMR and (B) ^29^Si-NMR for N12 in CDCl_3_. Figure S20. (A) ^1^H-NMR and (B) ^29^Si-NMR for HO7-6 in MeOH-*d*_4_. Figure S21. ^1^H-NMR for HO62-6 in MeOH-*d*_4_. Figure S22. (A) ^1^H-NMR and (B) ^29^Si-NMR for HO9 in MeOH-*d*_4_. Figure S23. (A) ^1^H-NMR and (B) ^29^Si-NMR for HO12 in MeOH-*d*_4_. Figure S24. ^13^C-NMR for a) 3-Vi, b) 9OEt, c) 9-Vi, d) N9 in CDCl_3_ and e) HO9 in MeOH*-d4*. Figure S25. Titration by copper (II) of (A) N12. (B) N6. Figure S26. Job Plots of (A) N12. (B) N6. Figure S27. Adsorption of Cu^2+^ into a thin film of HO62-6 coated on a silicone elastomer slab.

## Sample Availability

Samples are not available from the authors.
