# Supplementary material for: Chelating Silicone Dendrons: Trying to Impact Organisms by Disrupting Ions at Interfaces
Source: Molecules. 2022 Mar 14;27(6):1869. doi: 10.3390/molecules27061869 (PMC8954278; doi:10.3390/molecules27061869)
Supplement: Supplementary file 1 [file molecules-27-01869-s001.zip › molecules-1591803-supplementary.pdf]

Chelating Silicone Dendrons: Trying to Impact Organisms by Disrupting Ions at Interfaces

Miguel Melendez-Zamudio [1], Kevina Chavda [1] and Michael A. Brook [1,*]

Supporting Information

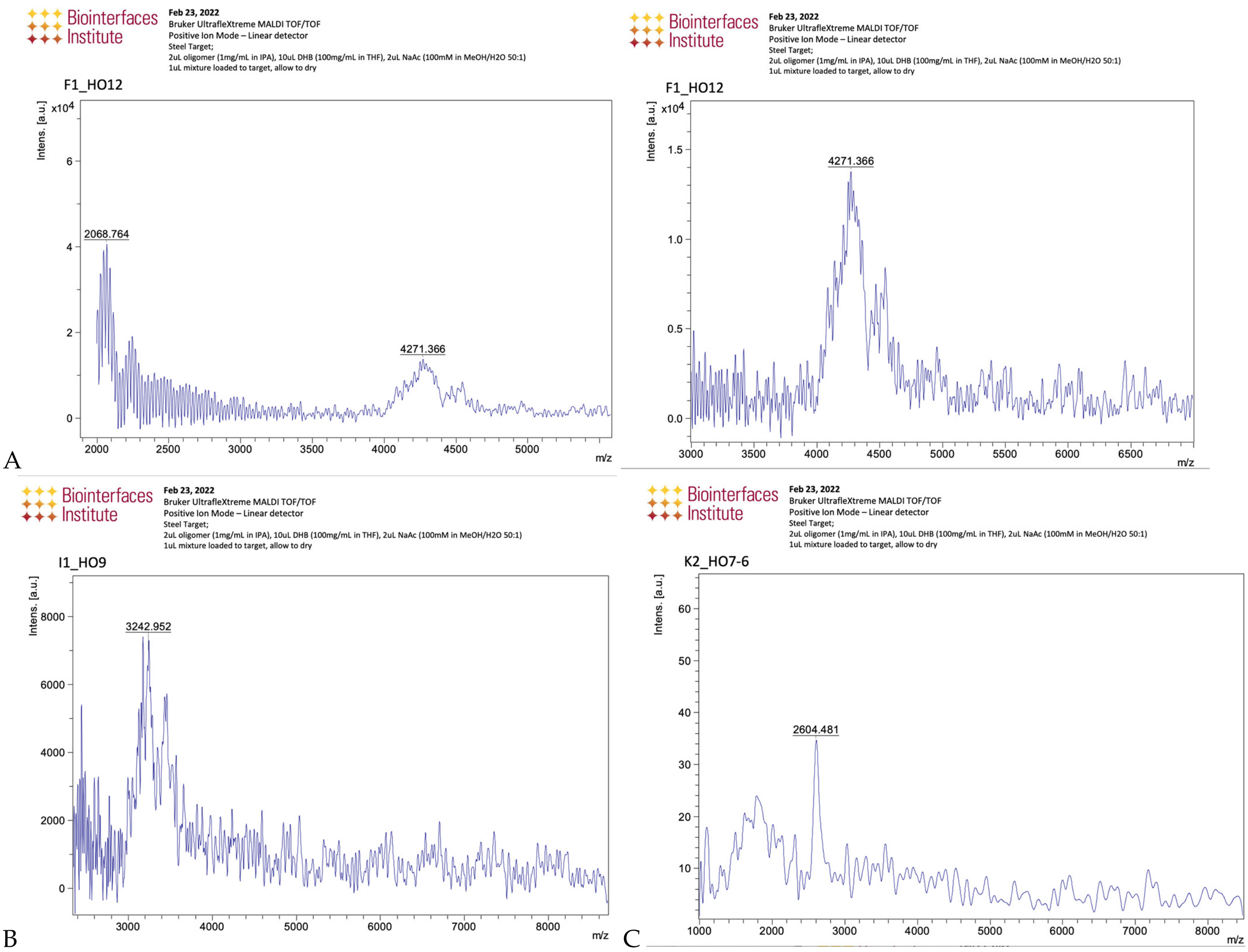

Figure S1. MALDI mass spectra (Bruker UltrafleXtreme MALDI TOF/TOF; 2 µL oligomer (1 mg/mL in IPA, (isopropanol)), 10µL DHB (2,5-dihydroxybenzoic acid, 100 mg/mL in THF), 2 µL NaOAc (100 mM in MeOH/H_2O 50:1) 1 µL mixture loaded to target, allowed to dry.) of A) **HO12**, B) **HO12**, and C) **HO7-6**.

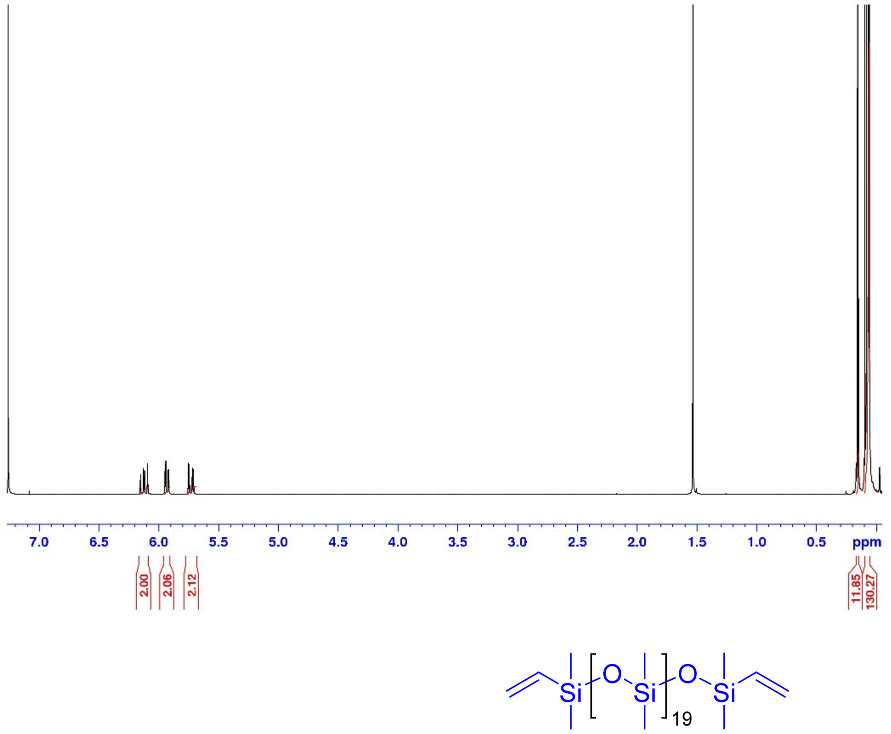

Figure S2. 1H NMR for **2-Vi** in $CDCl_3$.

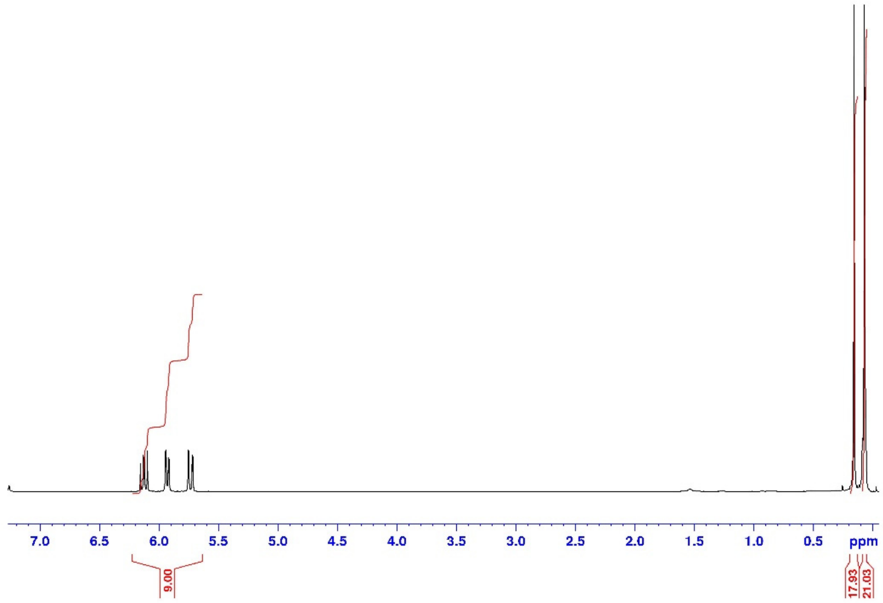

A

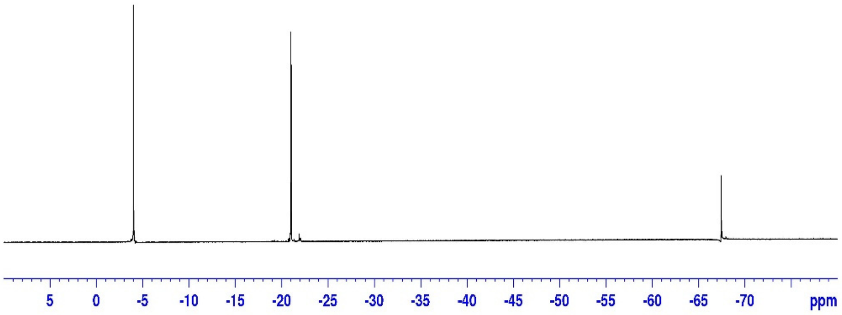

B

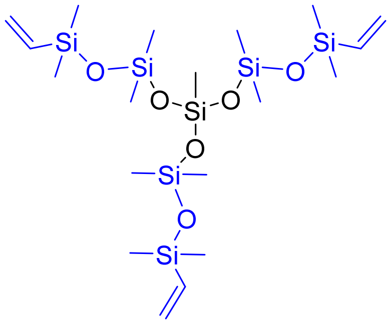

Figure S3. A) ^{1}H NMR and B) ^{29}Si NMR for **3-Vi** in $CDCl_3$.

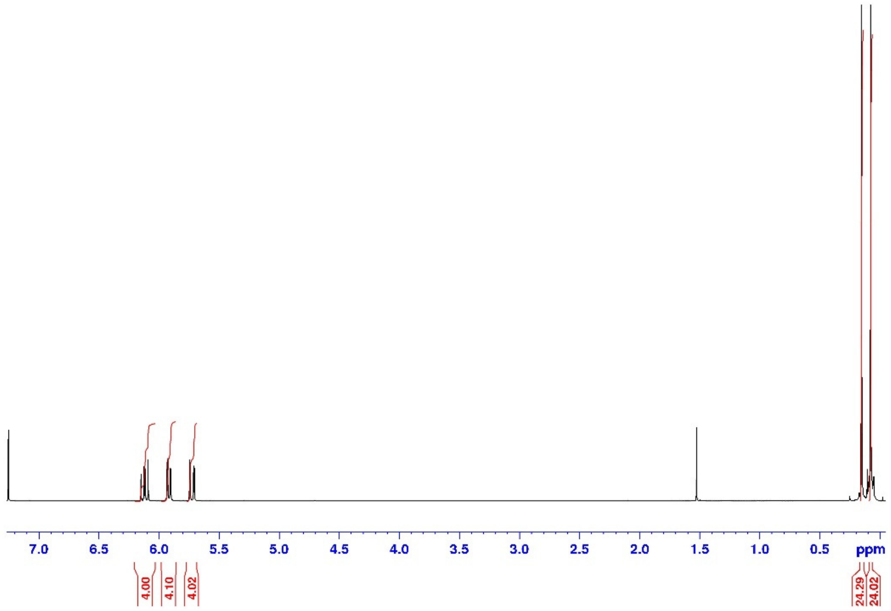

A

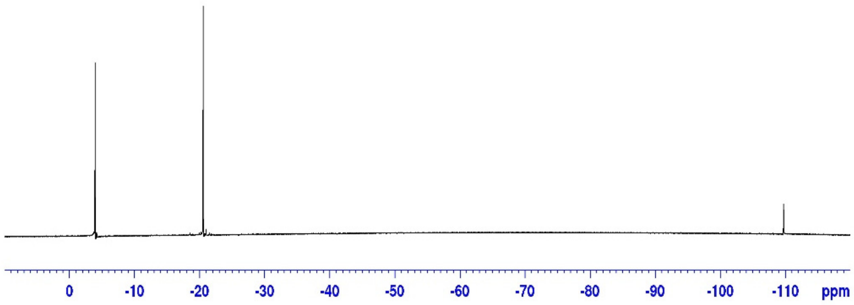

B

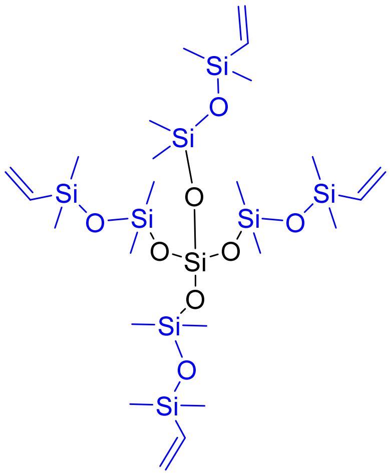

Figure S4. A) ^{1}H NMR and B) ^{29}Si NMR for **4-Vi** in $CDCl_3$.

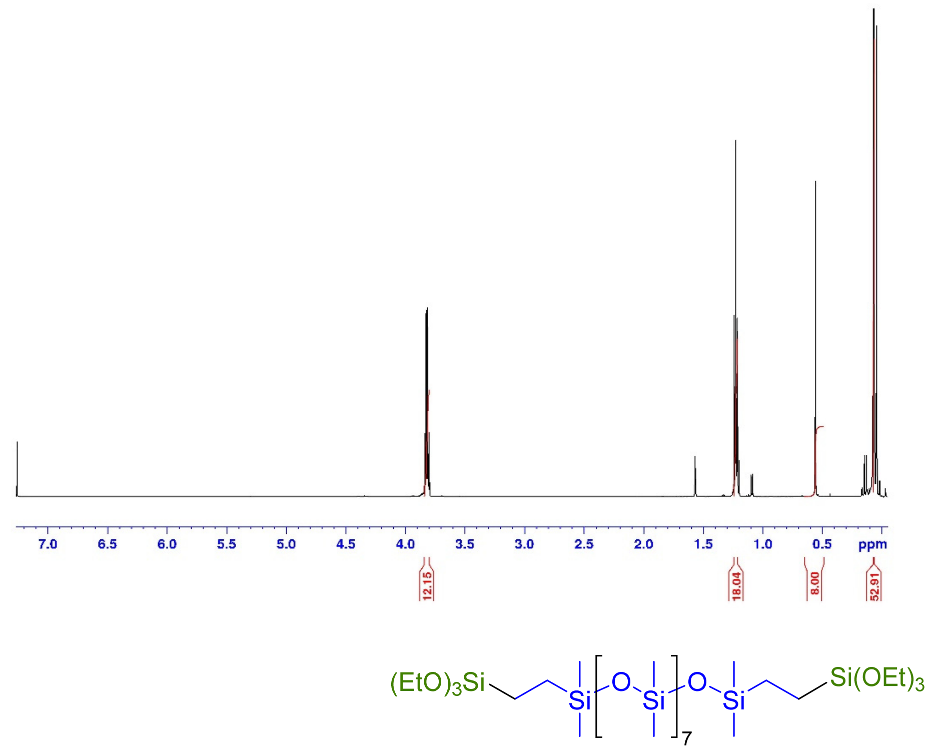

Figure S5. ^{1}H NMR for **7-6OEt** in $CDCl_3$.

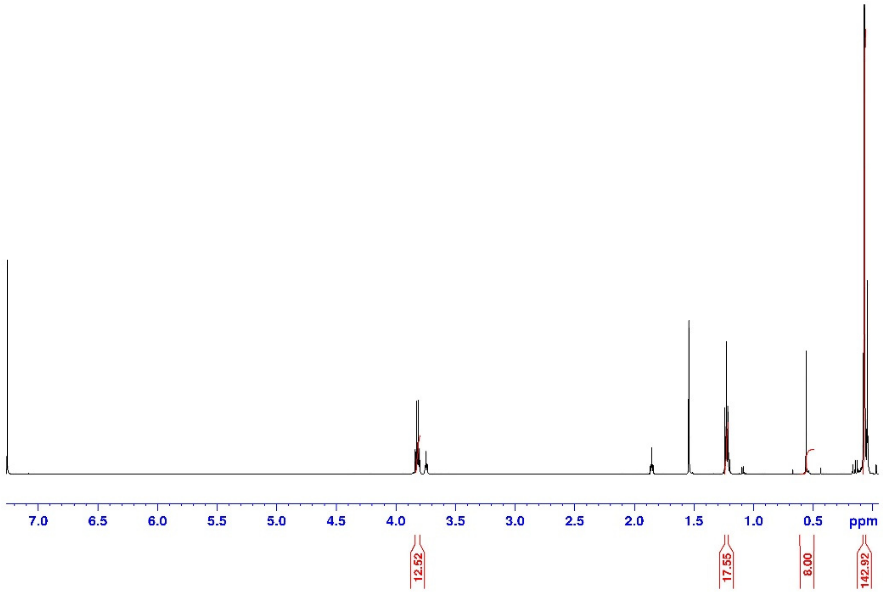

A

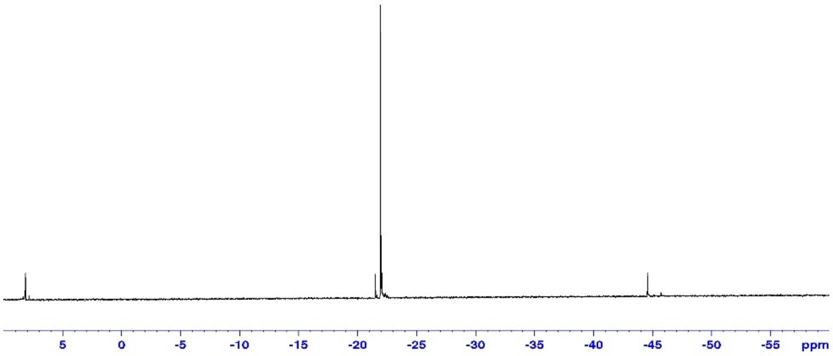

B

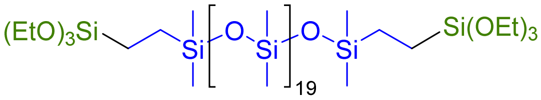

Figure S6. A) ^{1}H NMR and B) ^{29}Si NMR for **19-6OEt** in $CDCl_3$.

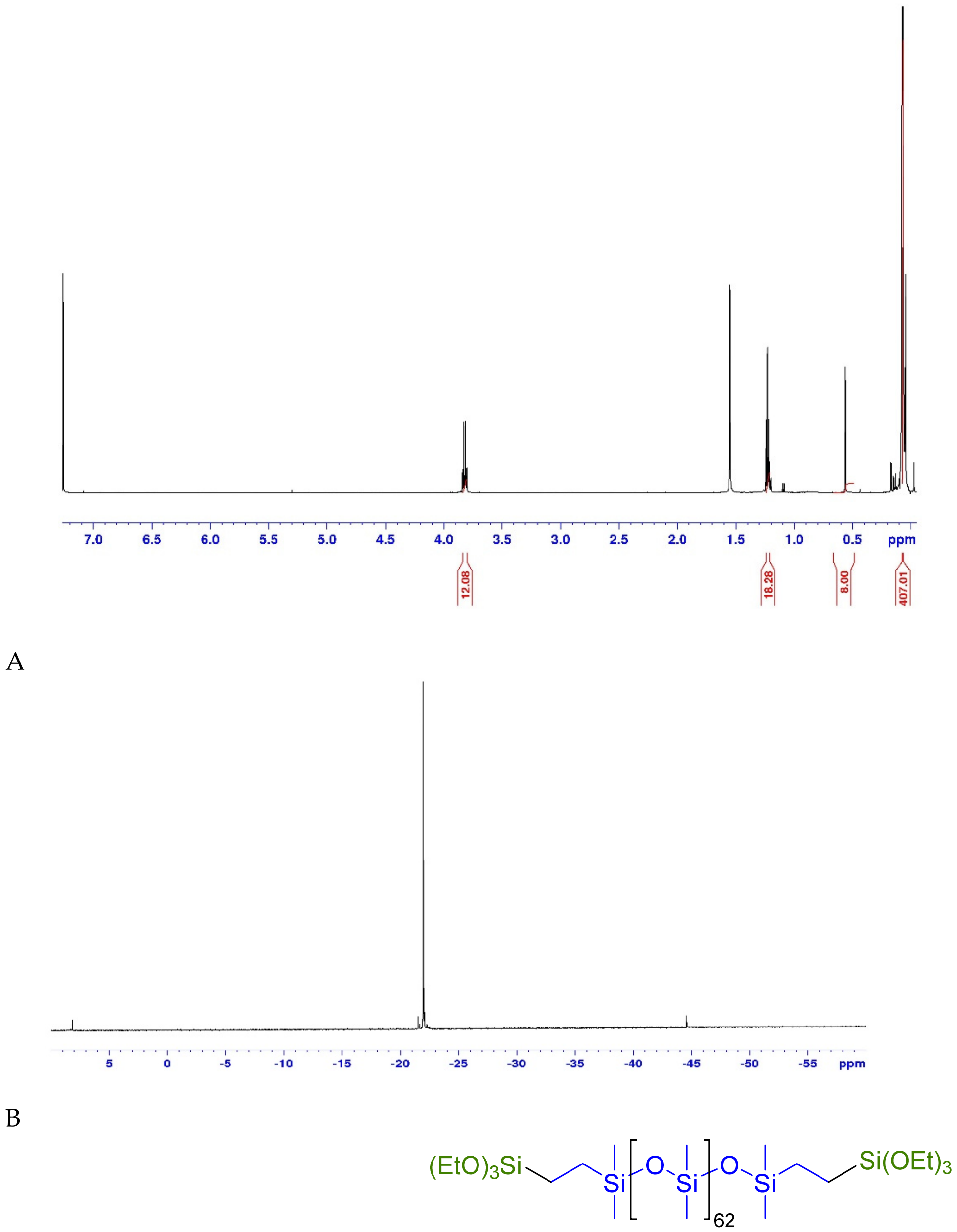

Figure S7. A) ^{1}H NMR and B) ^{29}Si NMR for **62-6OEt** in $CDCl_3$.

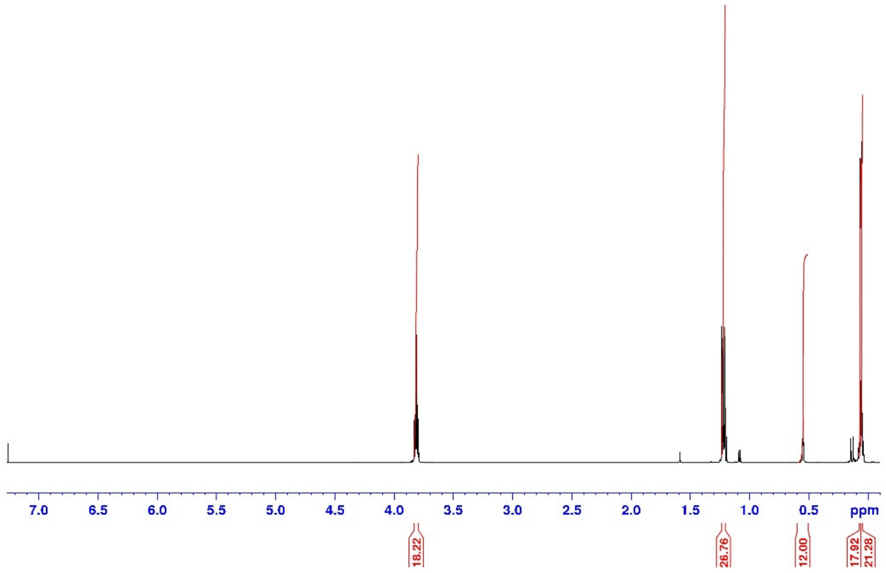

A

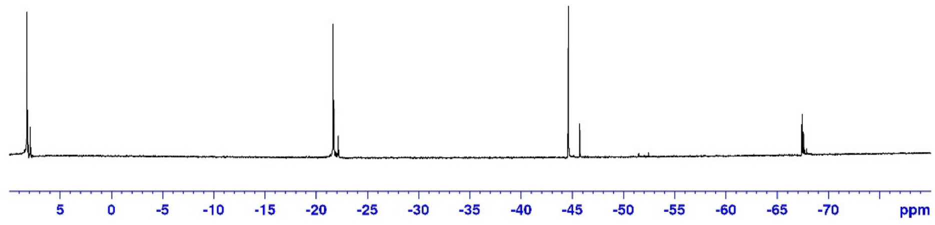

B

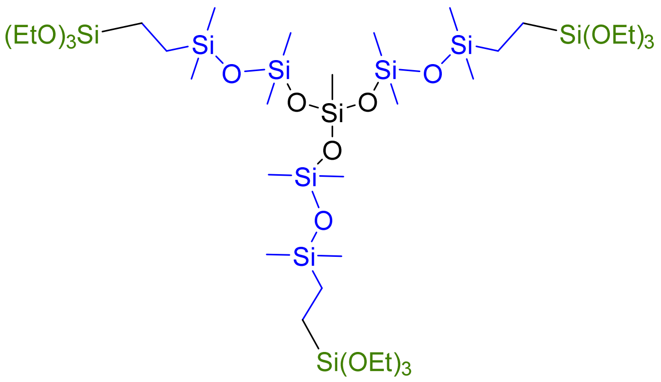

Figure S8. A) ^{1}H NMR and B) ^{29}Si NMR for **9OEt** in $CDCl_3$.

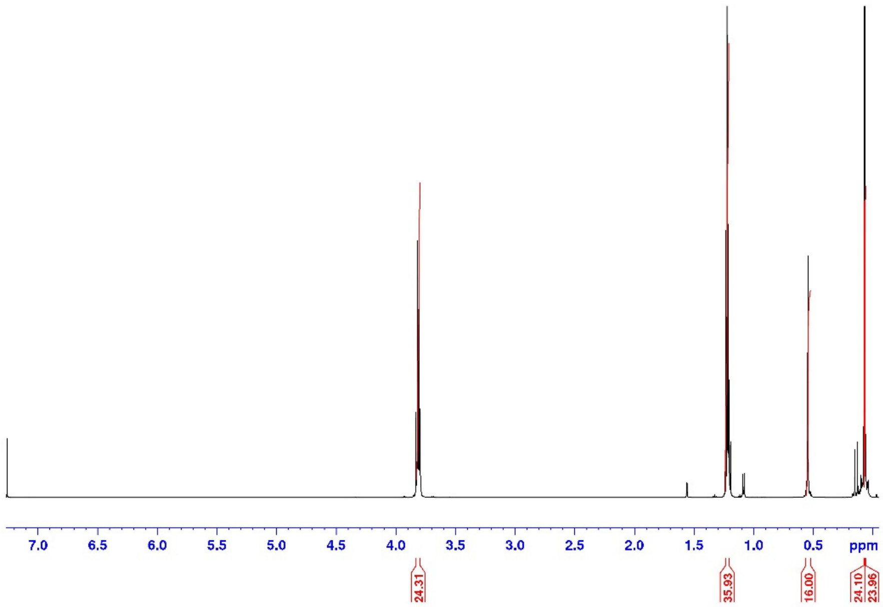

A

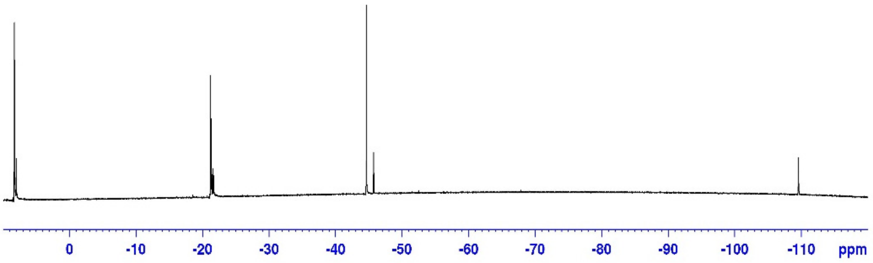

B

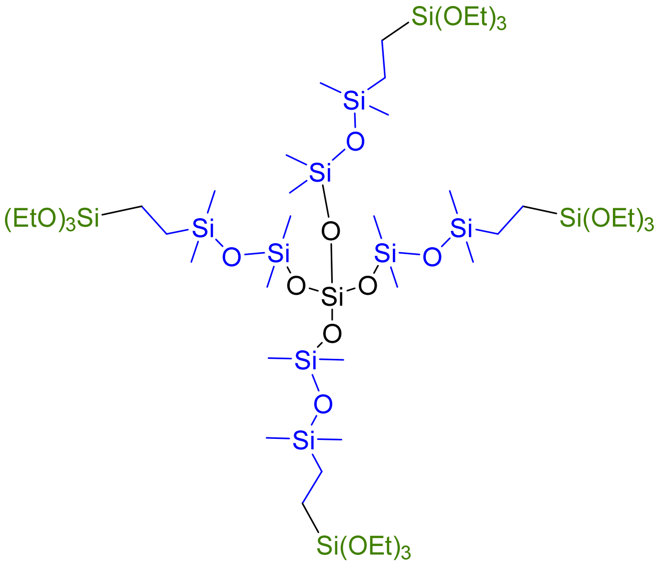

Figure S9. A) ^{1}H NMR and B) ^{29}Si NMR for **12OEt** in $CDCl_3$.

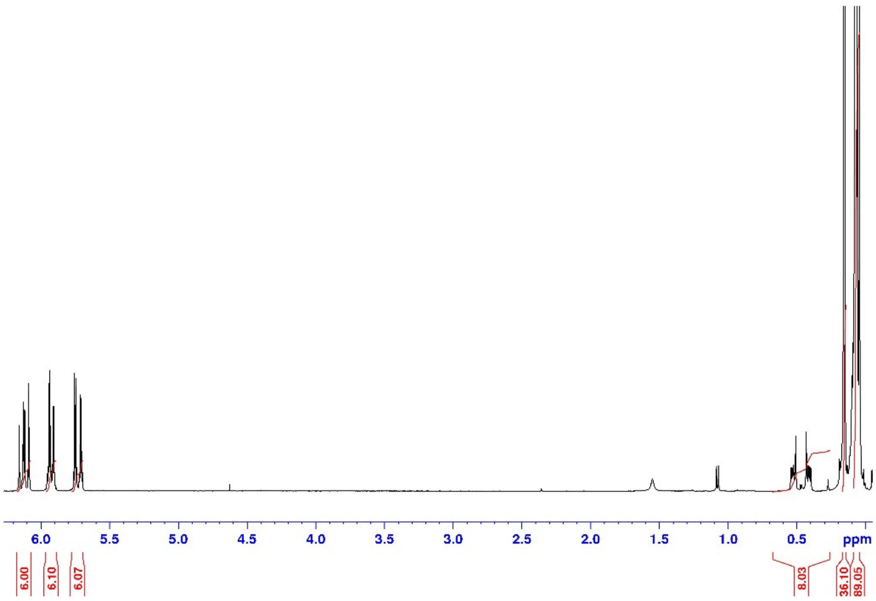

A

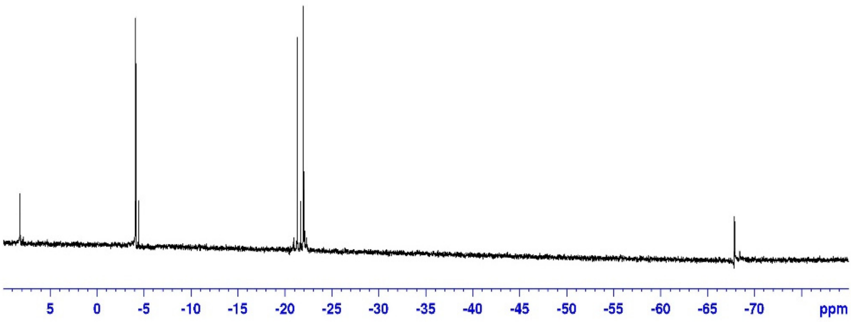

B

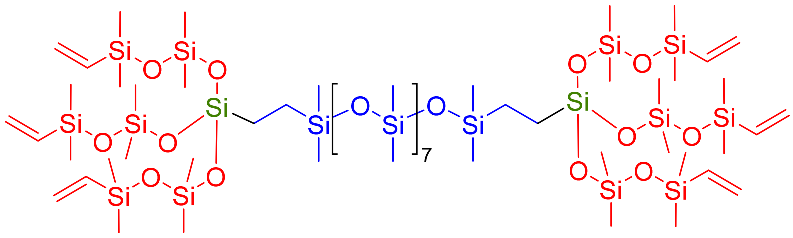

Figure S10. A) ^{1}H NMR and B) ^{29}Si NMR for **7-6Vi** in $CDCl_3$.

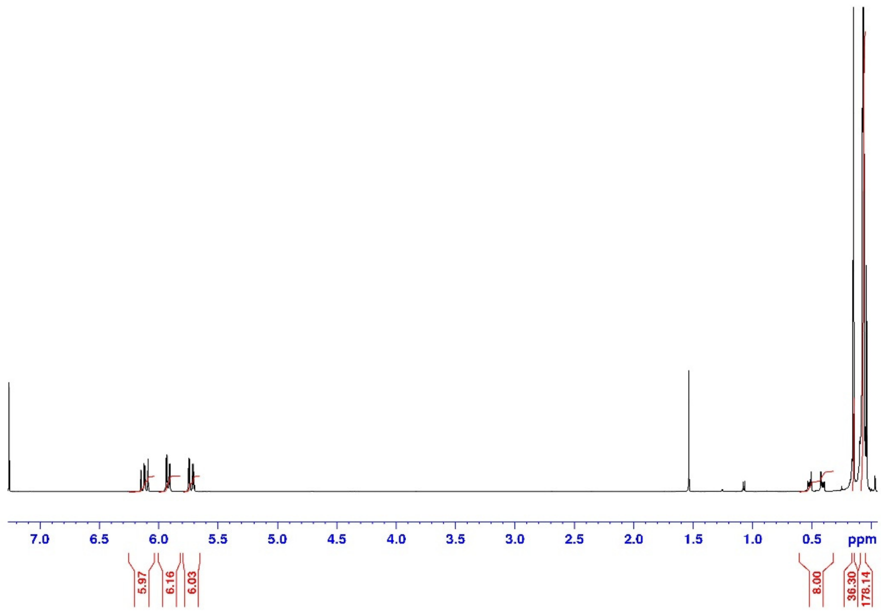

A

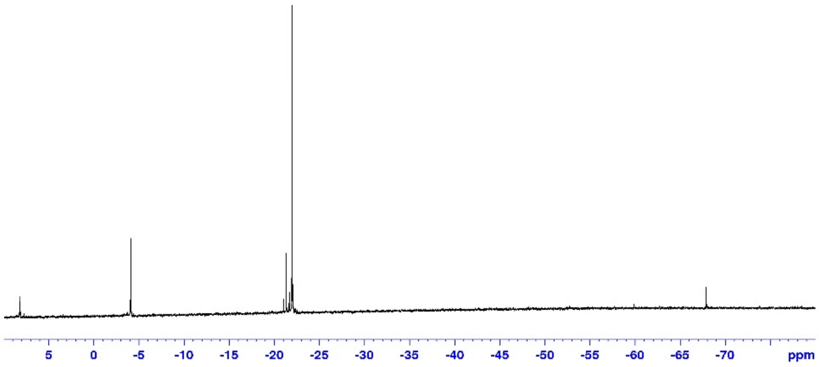

B

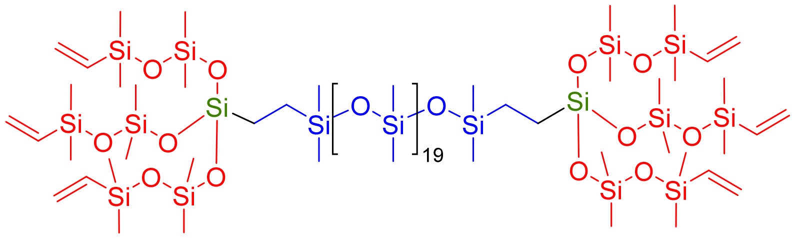

Figure S11. A) ^{1}H NMR and B) ^{29}Si NMR for **19-6OVi** in $CDCl_3$.

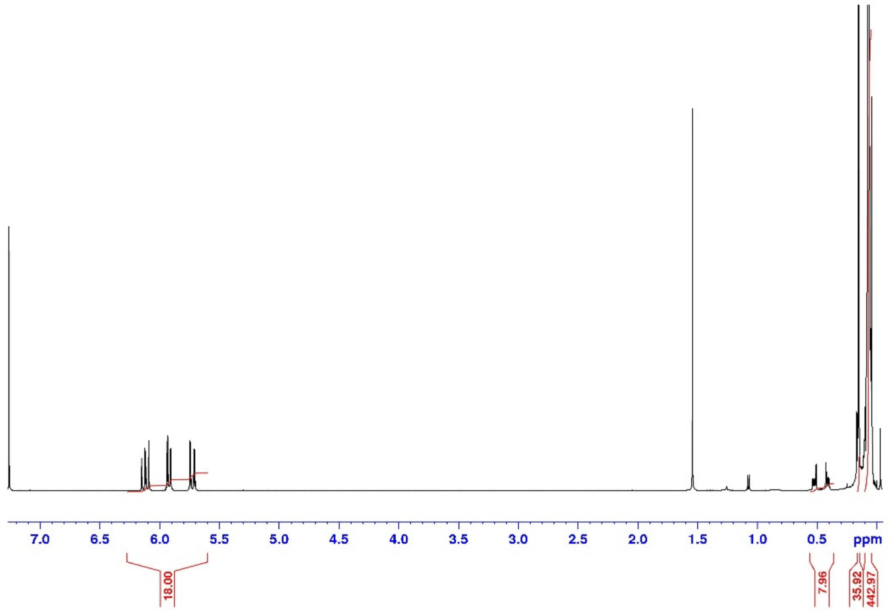

A

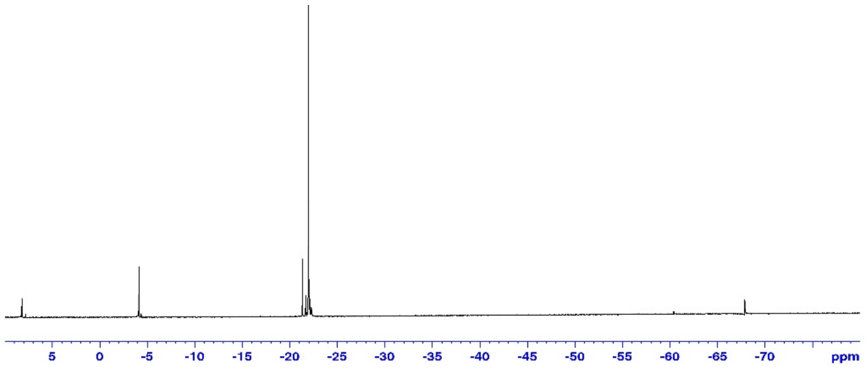

B

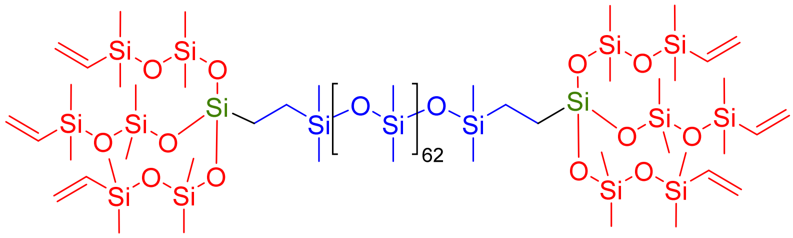

Figure S12. A) ¹H NMR and B) ²⁹Si NMR for **62-6Vi** in CDCl3.

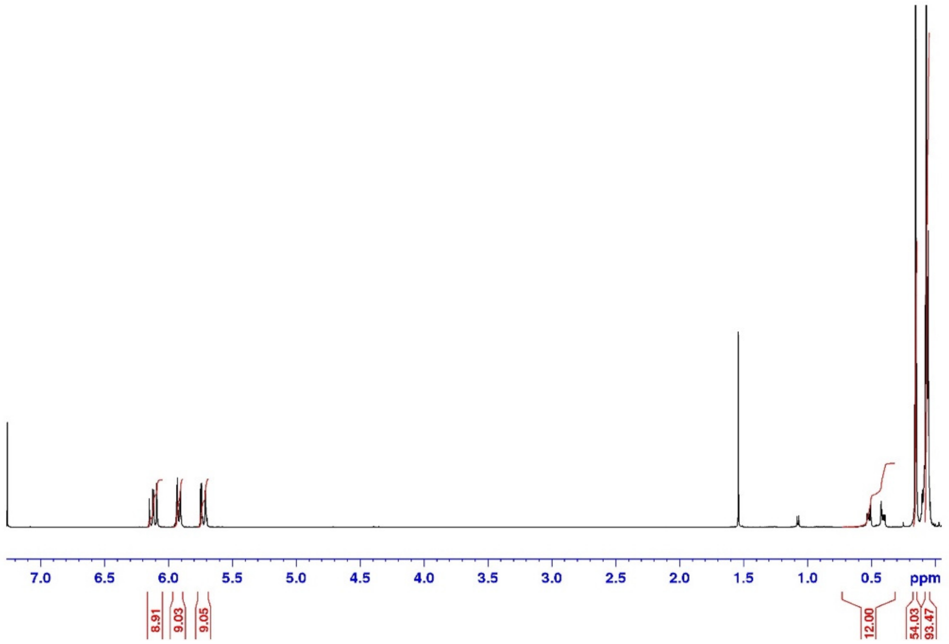

A

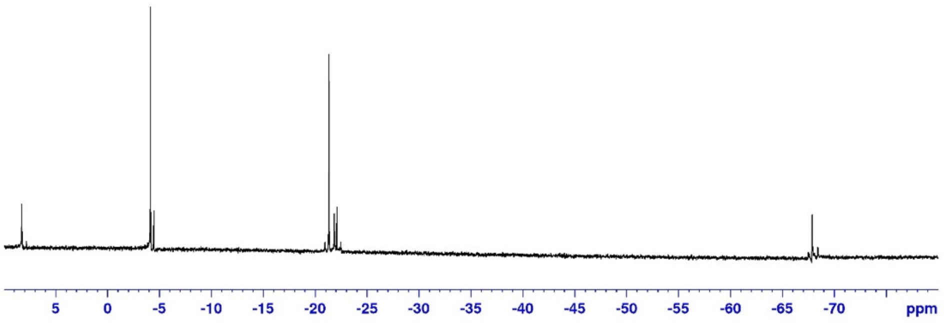

B

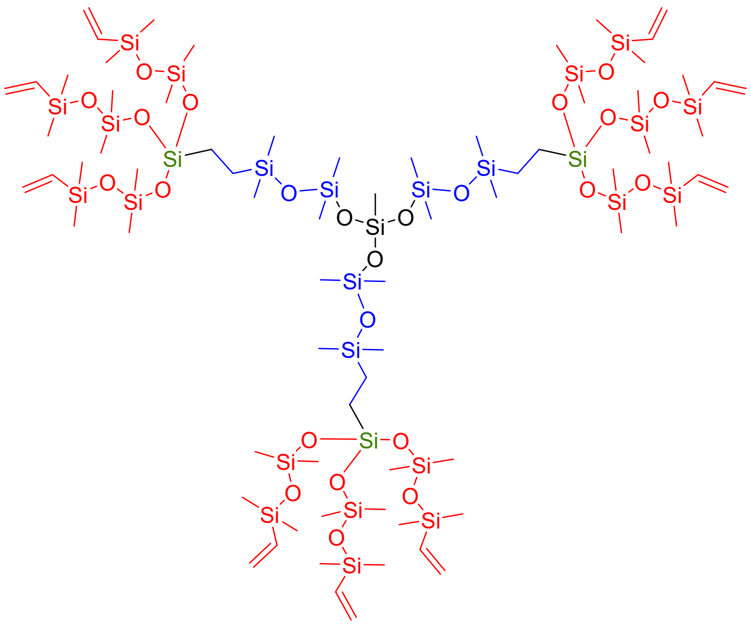

Figure S13. A) ^{1}H NMR and B) ^{29}Si NMR for **9Vi** in $CDCl_3$.

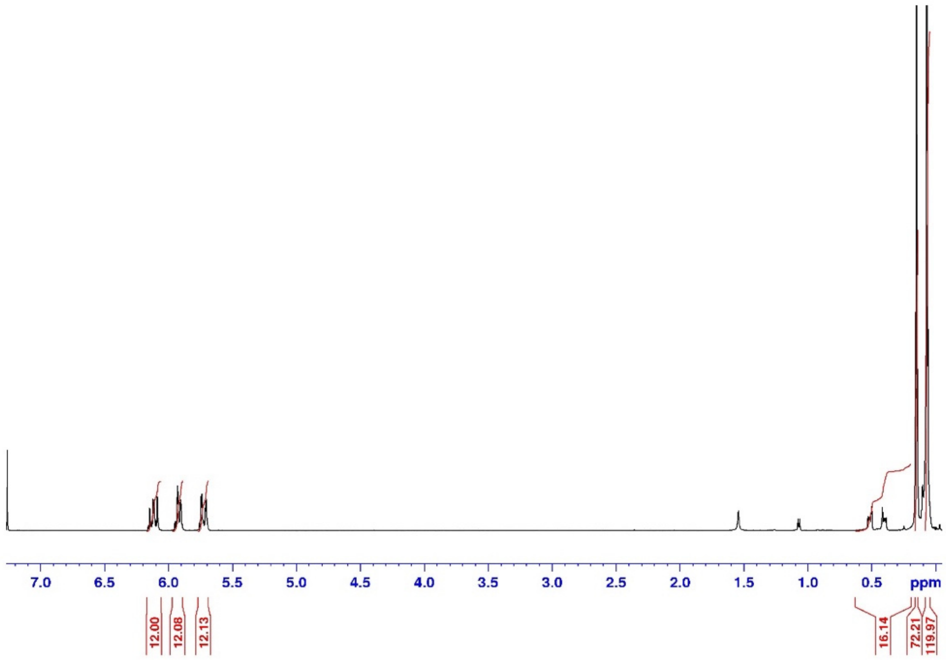

A

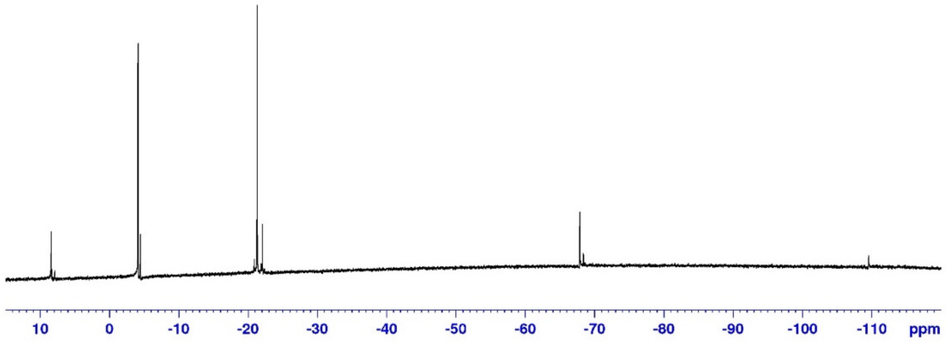

B

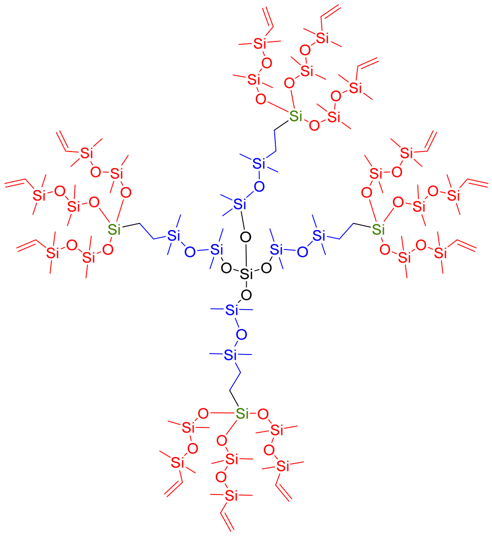

Figure S14. A) ^{1}H NMR and B) ^{29}Si NMR for **12Vi** in $CDCl_3$.

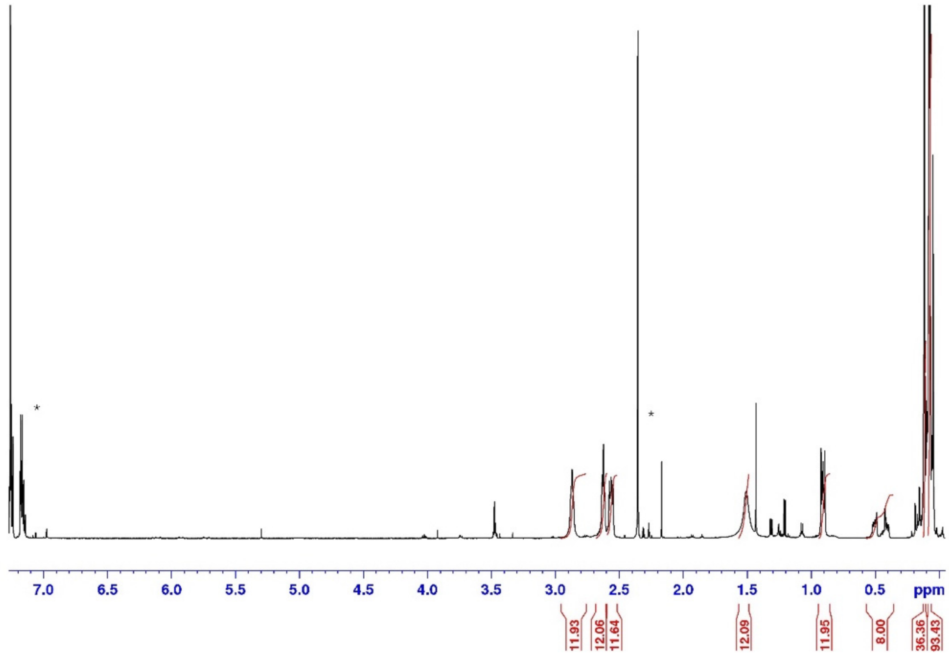

A

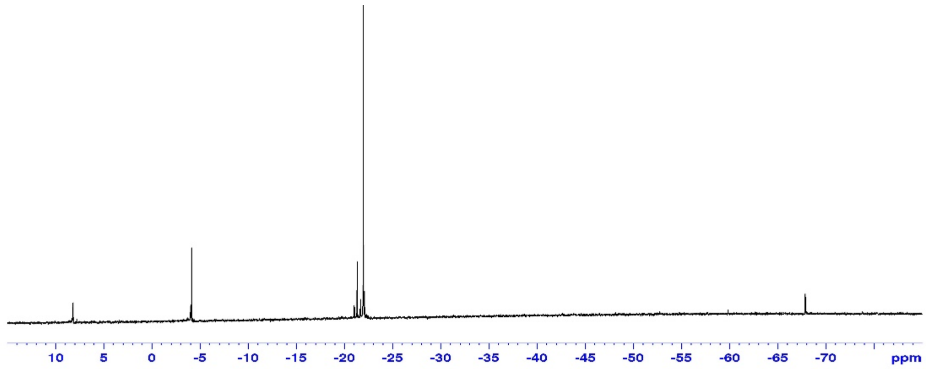

B

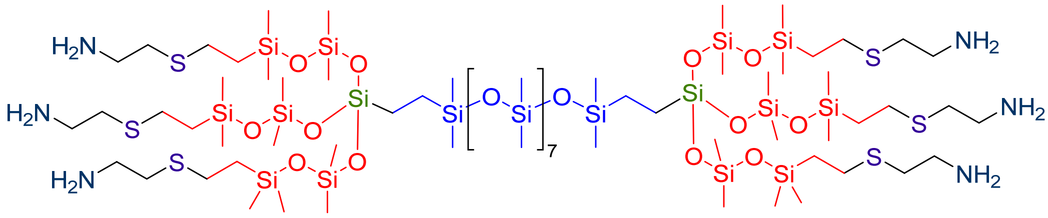

Figure S15. A) ^{1}H NMR and B) ^{29}Si NMR for **N 7-6** in $CDCl_3$.

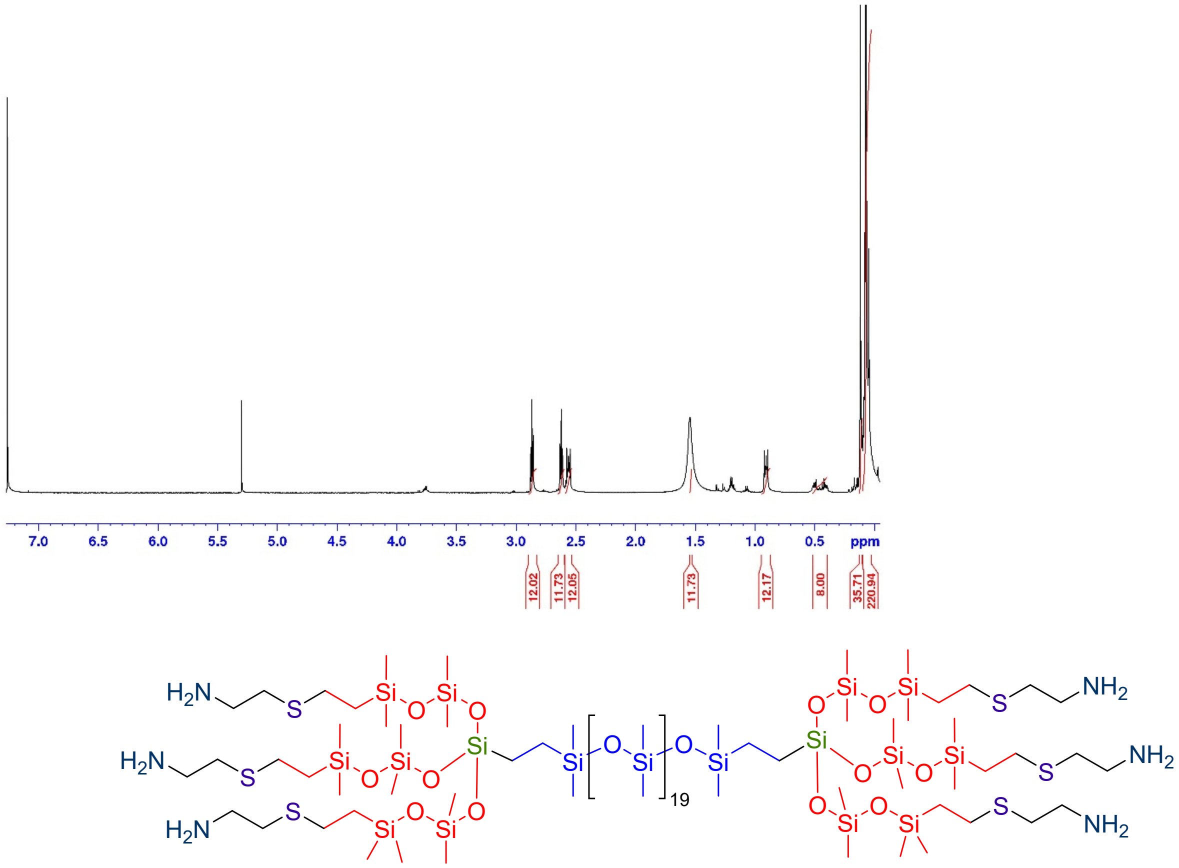

Figure S16. ^{1}H NMR for **N 19-6** in $CDCl_3$.

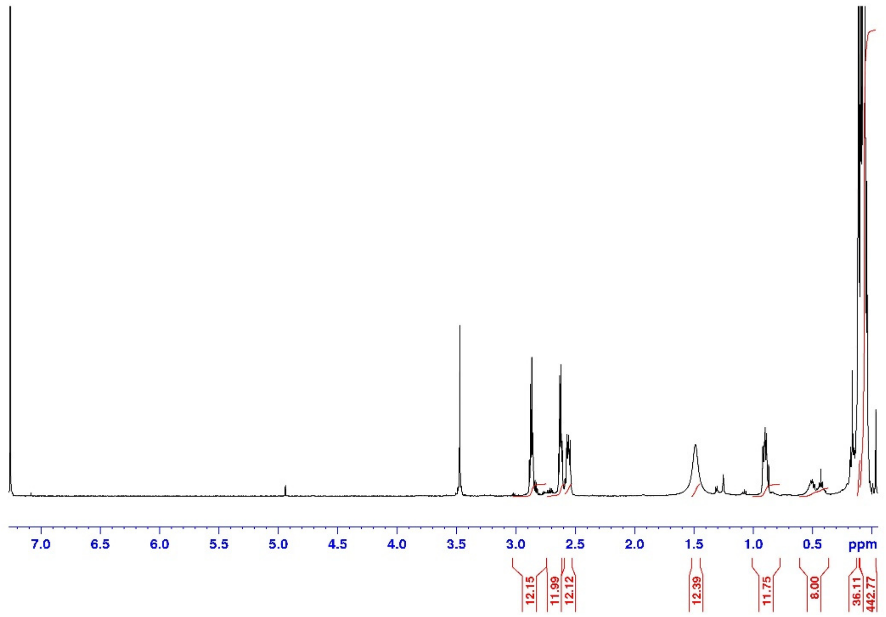

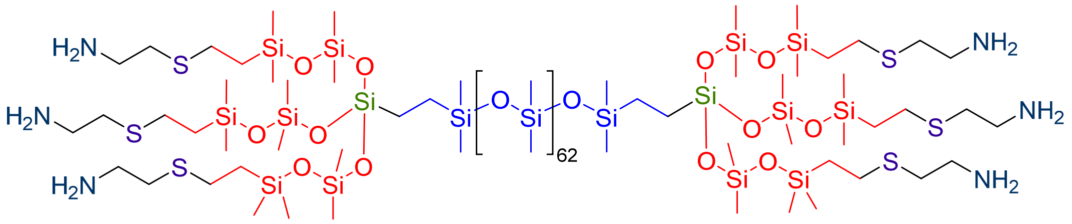

Figure S17. ^{1}H NMR for **N 62-6** in CDCl3.

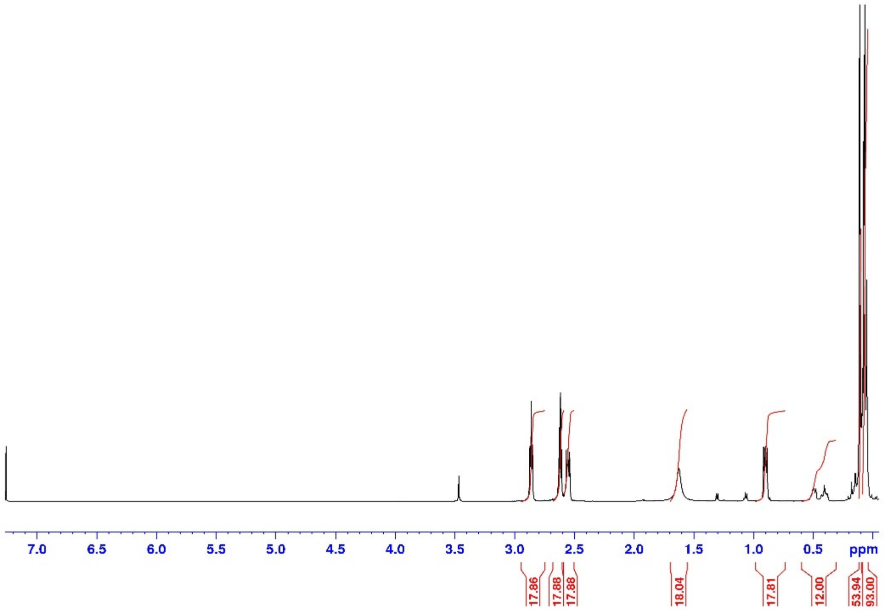

A

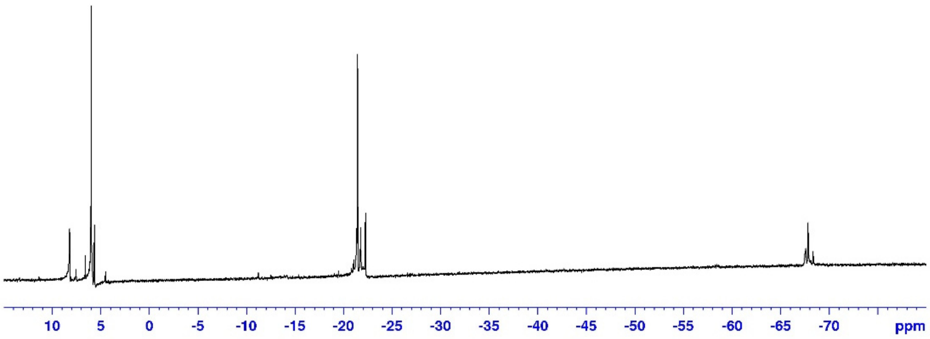

B

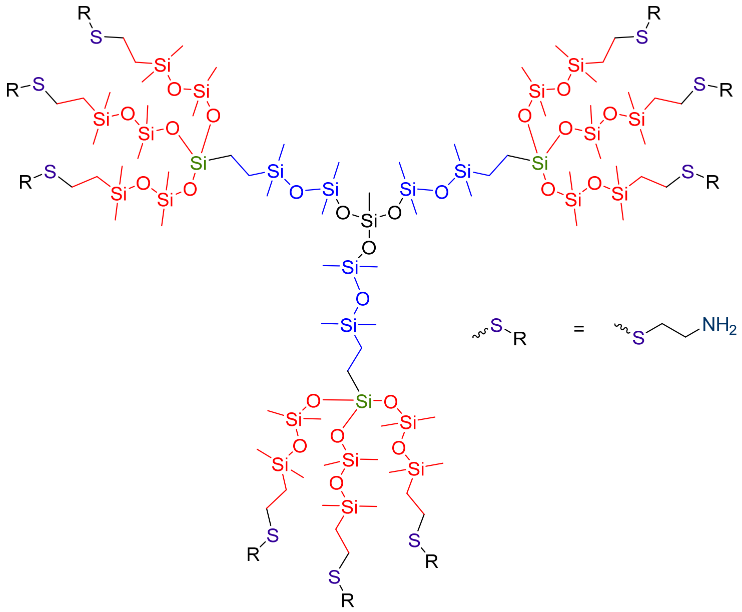

Figure S18. A) ^{1}H NMR and B) ^{29}Si NMR for **N9** in $CDCl_3$.

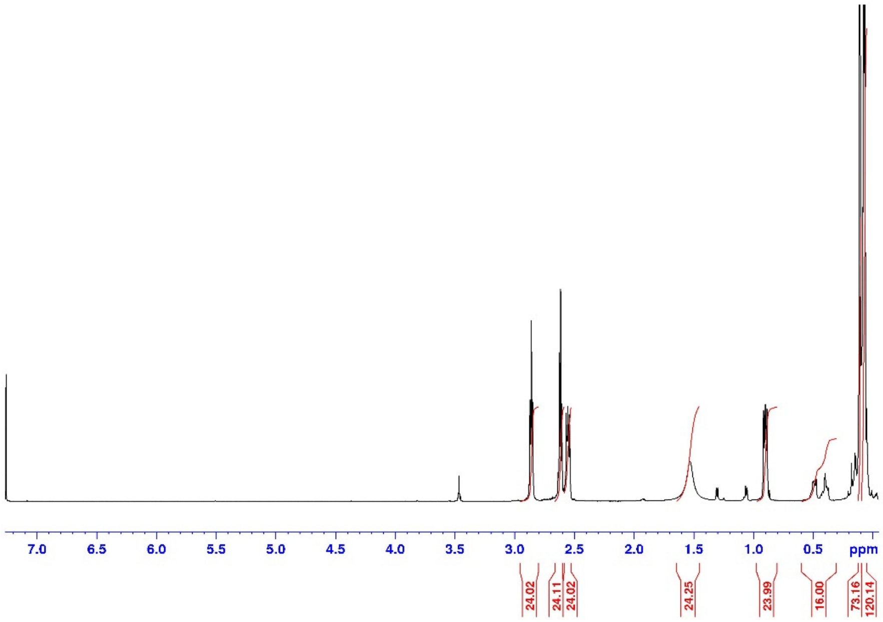

A

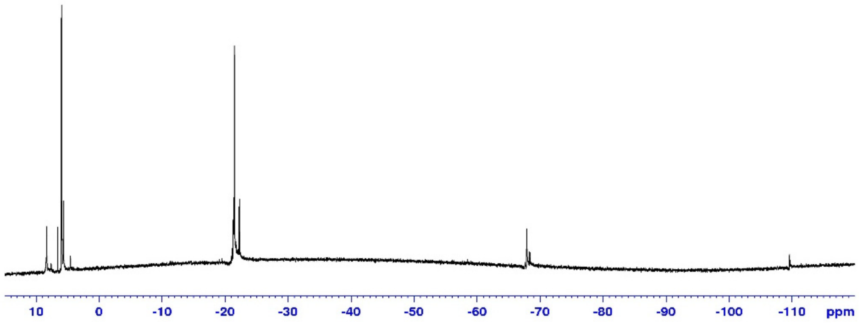

B

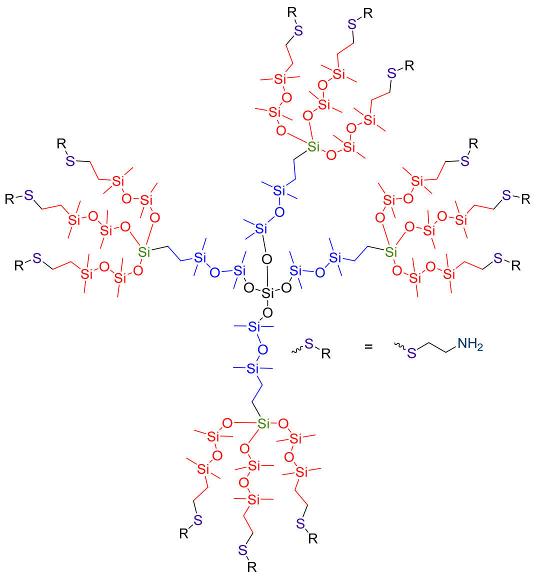

Figure S19. A) ^{1}H NMR and B) ^{29}Si NMR for **N12** in $CDCl_3$.

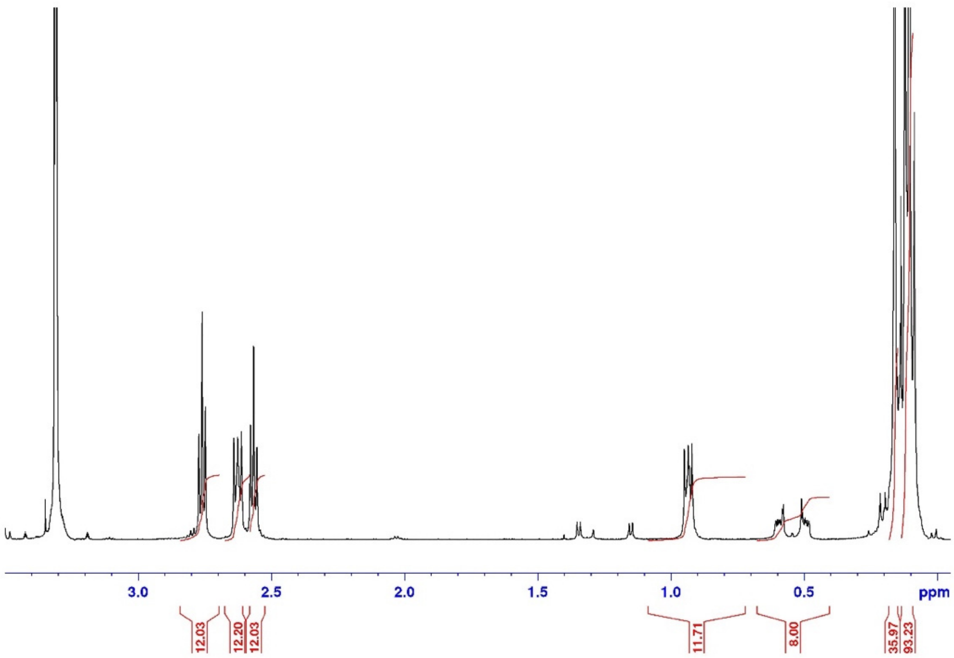

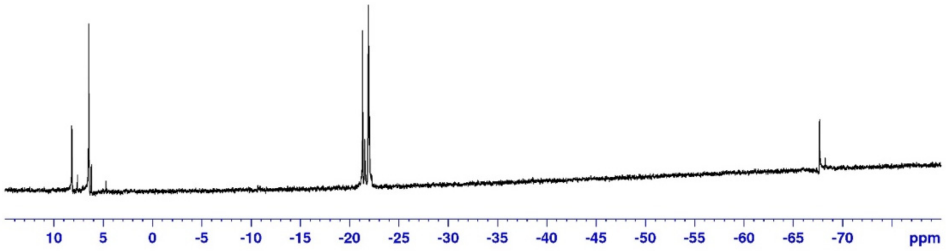

B

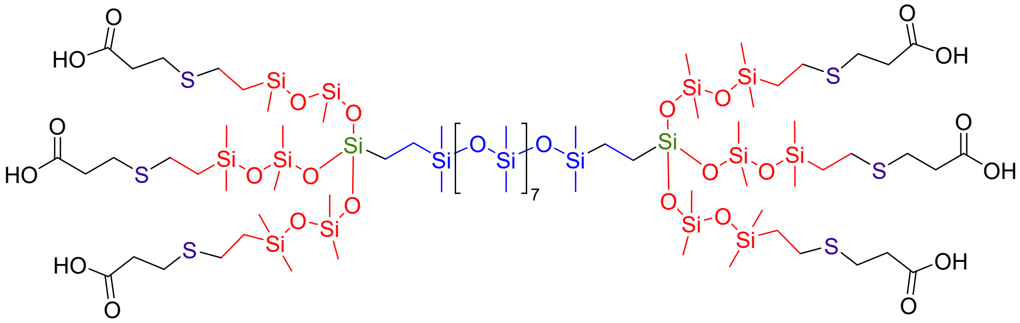

Figure S20. A) ^{1}H NMR and B) ^{29}Si NMR for **HO7-6** in MeOH-*d*$_4$.

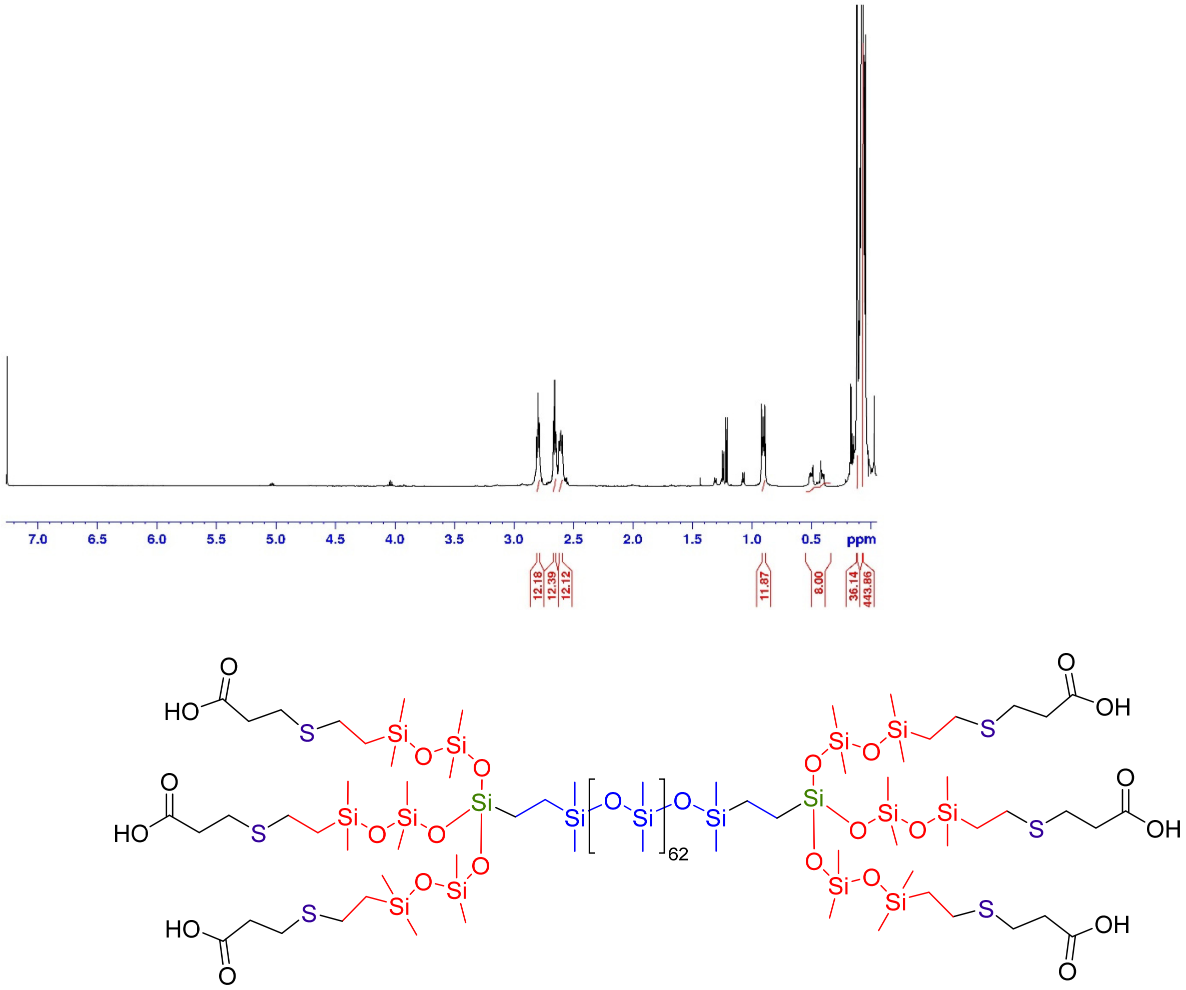

Figure S21. ^{1}H NMR for **HO62-6** in $CDCl_3$..

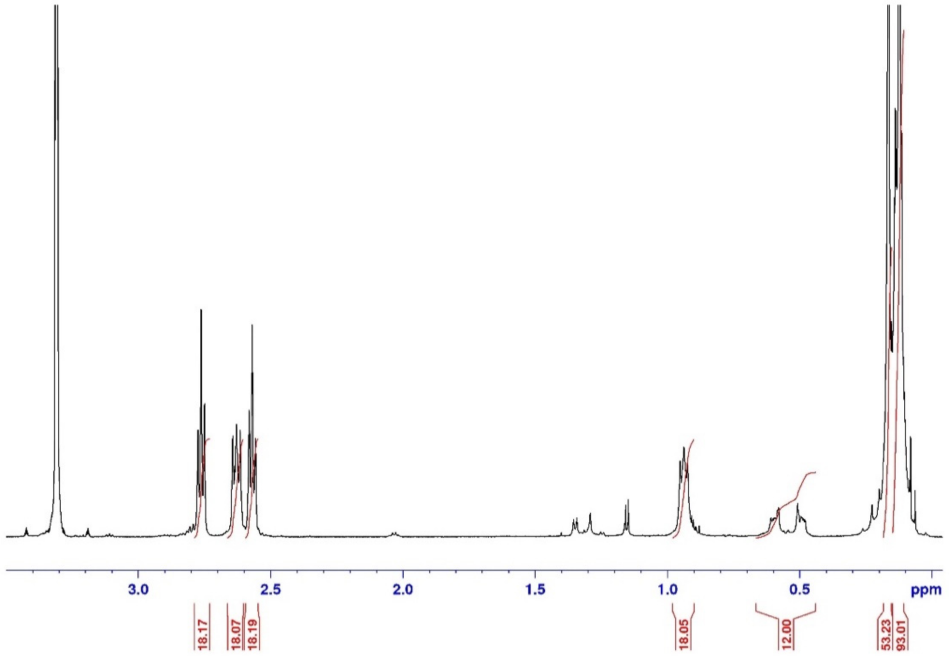

A]

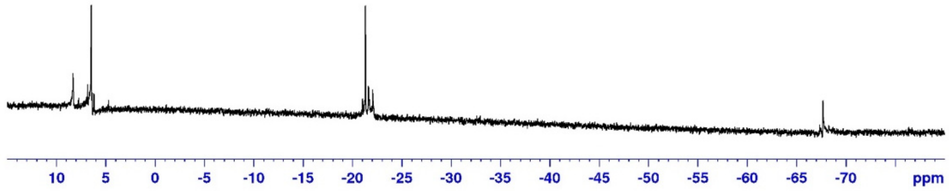

B

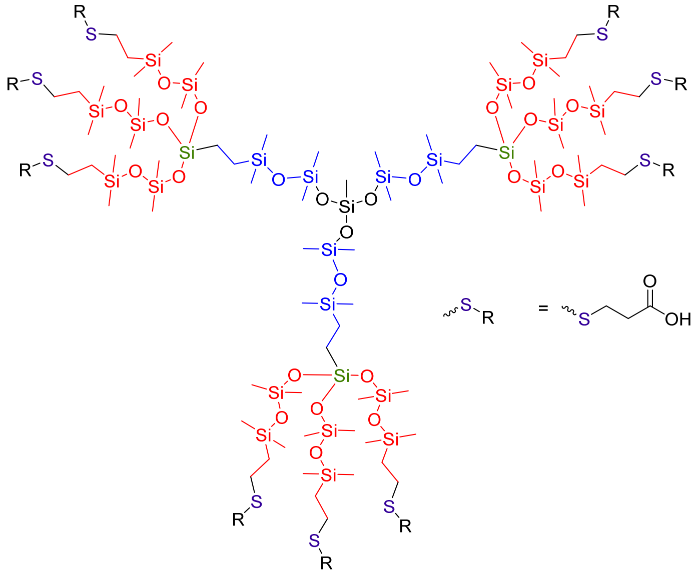

Figure S22. A) ^{1}H NMR and B) ^{29}Si NMR for **HO9** in MeOH-d_4.

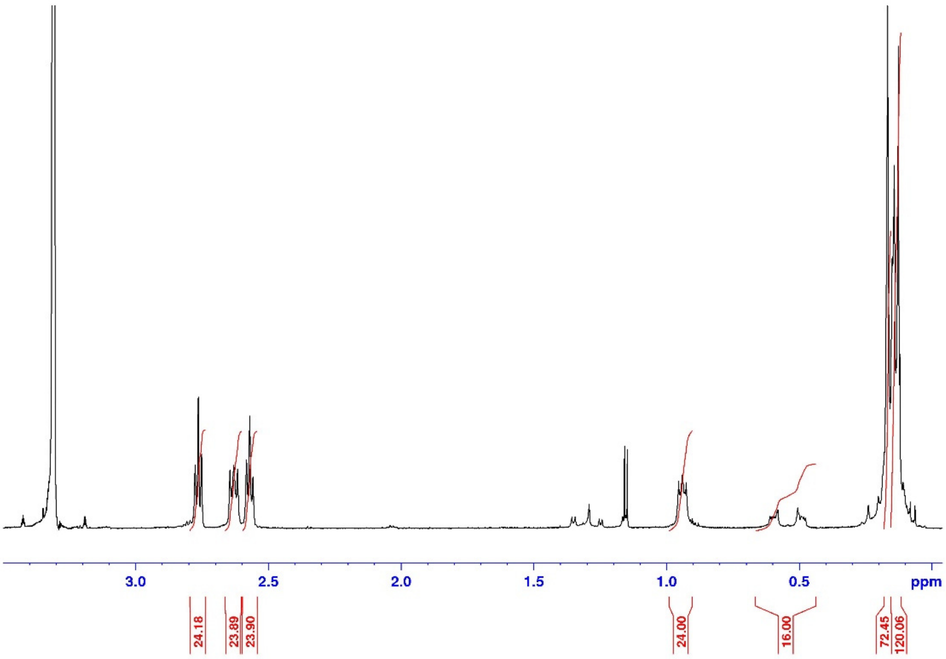

A

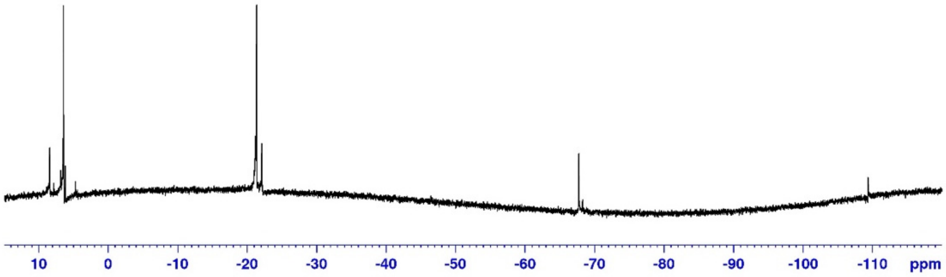

B

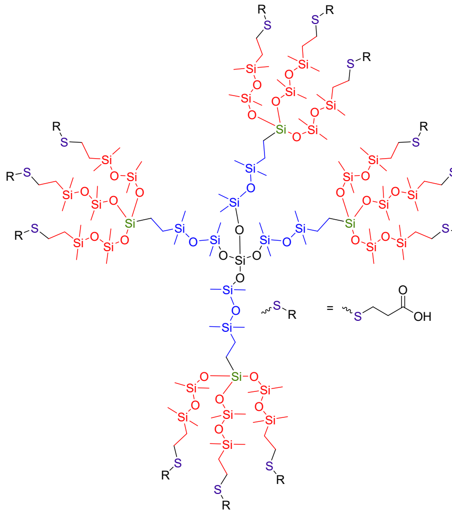

Figure S23. A) ^{1}H NMR and B) ^{29}Si NMR for **HO12** in MeOH-d_4.

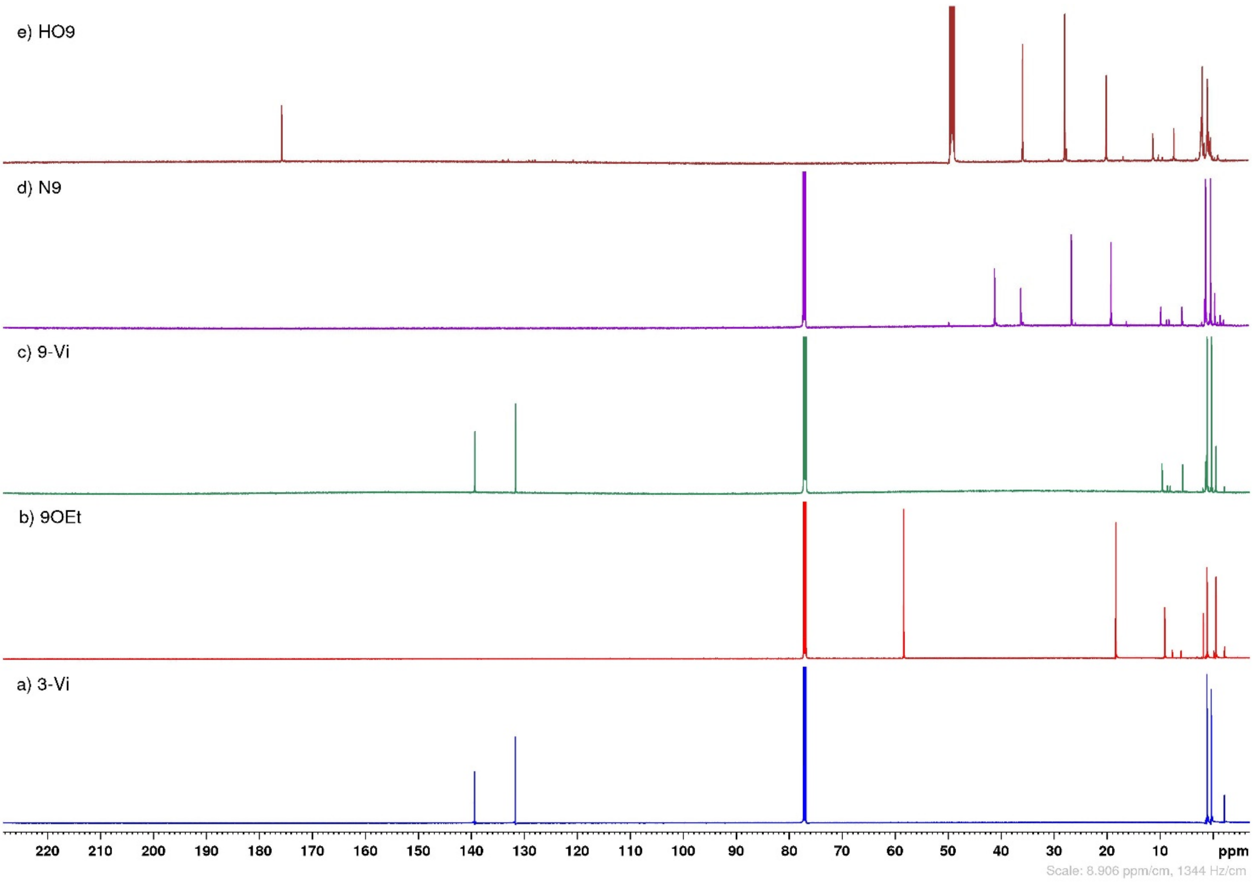

Figure S24. ^{13}C NMR for a) **3-Vi**, b) **9OEt**, c) **9-Vi**, d) **N9** in $CDCl_3$ and e) **HO9** in MeOH-*d4*

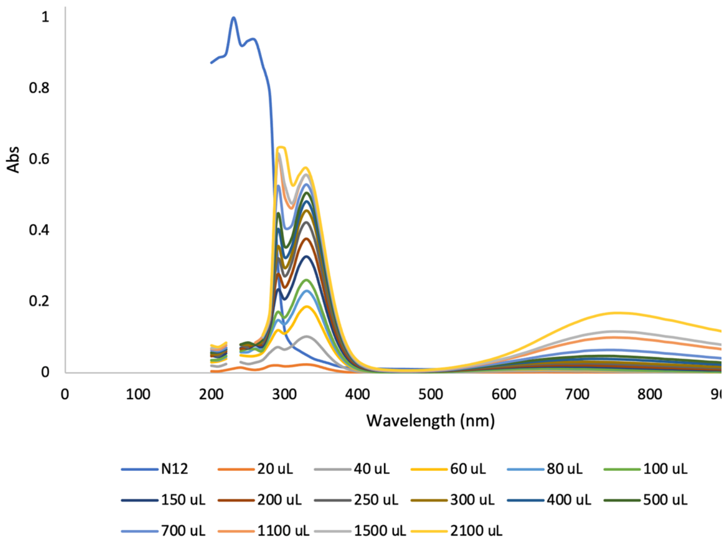

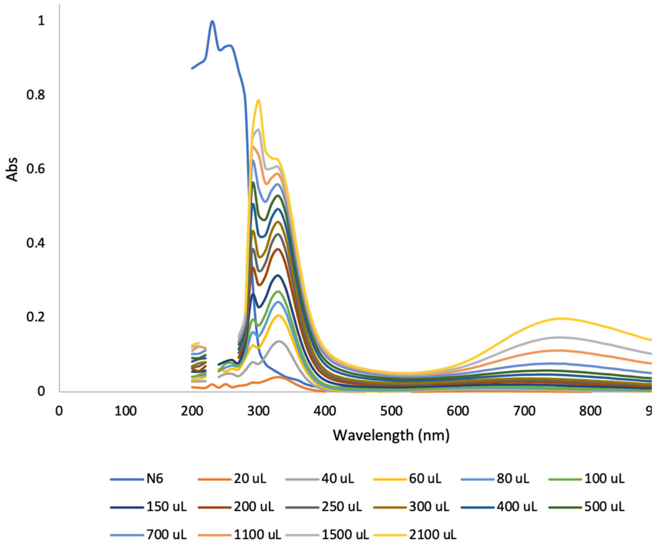

Figure S25. Titration by copper (II) of (a) **N12**. (b) **N6**.

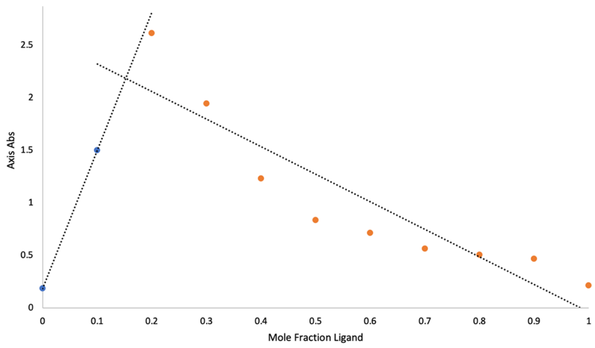

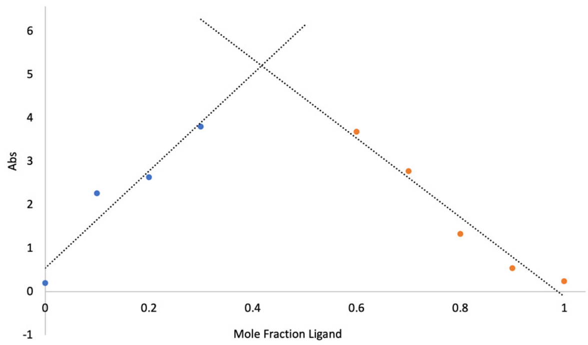

Figure S26. Job Plots of (a) **N12**. (b) **N6**.

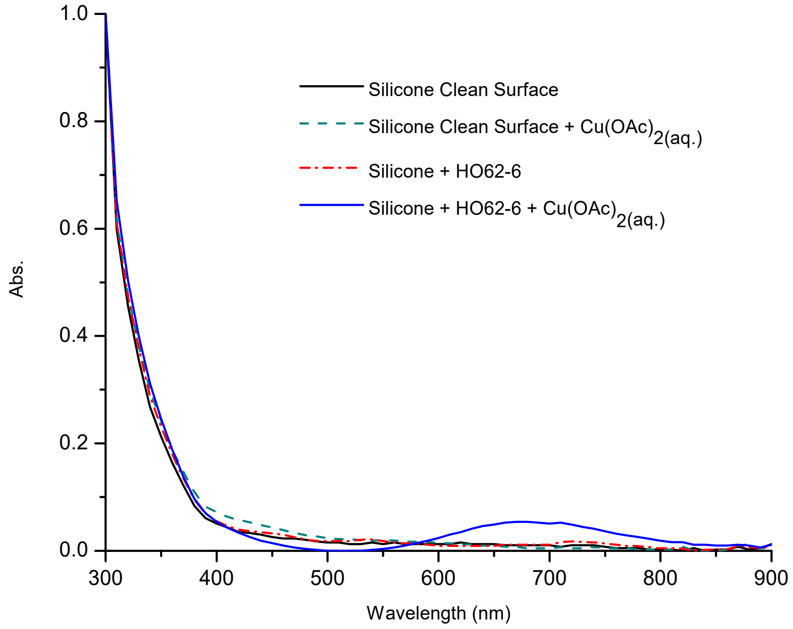

Figure S27. Adsorption of Cu^{2+} into a thin film of **HO62-6** coated on a silicone elastomer slab.
